# Combined impacts of global changes on biodiversity across the USA

**DOI:** 10.1038/srep11828

**Published:** 2015-07-07

**Authors:** C. Bellard, C. Leclerc, F. Courchamp

**Affiliations:** 1 Genetics, Evolution & Environment, Div Biosciences, Center for Biodiversity, Environment & Research, University College of London; 2 Ecologie, Systématique & Evolution, UMR CNRS 8079, Univ. Paris-Sud, F-91405 Orsay Cedex, France; 3 Department of Ecology and Evolutionary Biology and Center for Tropical Research, Institute of the Environment and Sustainability, University of California Los Angeles, CA 90095, USA

## Abstract

Most studies of the effects of global changes on biodiversity focus on a single threat, but multiple threats lead to species extinction. We lack spatially explicit assessments of the intensity of multiple threats and their impacts on biodiversity. Here, we used a novel metric of cumulative threats and impacts to assess the consequences of multiple threats on 196 endemic species across the USA. We predict that large areas with high cumulative impact scores for amphibians, birds, mammals, and reptiles will be concentrated in the eastern part of the USA by the 2050 s and 2080 s. These high cumulative impact values are due mainly to the presence of invasive species, climate change, cropland and pasture areas; additionally, a significant proportion of endemic species are vulnerable to some of these threats where they occur. This analysis provides a useful means of identifying where conservation measures and monitoring programs that should consider multiple threats should be implemented in the future.

Current threats have greatly accelerated the rate at which extinctions occur^1^. In general, multiple threats, such as habitat destruction, overexploitation, climate change, and invasive species, lead to an increase in species extinction risks^2^. The combined impacts of multiple threats are also diminishing the capacity of natural systems to cope with the effects of these changes. Yet over the past decade most studies have assessed the future spatial distribution of these threats individually^3^,^4^. This approach can be ineffective and ecologically misleading, because cumulative impacts of different threats are not considered. In addition, most of the conservation measures that are based on such studies consider a subset of threats, which may be of little benefit if other threats remain unaddressed. Consequently, assessing the number, the spatial distribution and the intensity of all major threats has been recognized as a priority for research and a critical component in conservation prioritization. However, focusing on the spatial distribution of threats is insufficient to ensure long-term conservation of species, because not all species are equally sensitive or vulnerable to these threats. Therefore, we also need to consider the richness and spatial distribution of the biodiversity that is potentially vulnerable to each of these threats. In response to this need, we adapted an index to quantify and map the cumulative impacts of global changes on terrestrial biodiversity. Previous studies of cumulative human impacts have used publicly available data on ecosystem impact and stressor intensity to estimate impacts on marine or freshwater ecosystems (e.g.^5^,^6^,^7^,). They calculated vulnerability weights of each habitat-stressor combination. However, these weights were based on expert judgments, risking potential biases due to subjective influences^8^. They also assumed that the weight given to stressors accurately represented their effects on ecosystems across entire regions^8^. Here, we calculated cumulative impact scores for threats specific to each taxonomic group considered, accounting for the unique vulnerability of each taxonomic group to each threat per pixel. This approach allowed us to identify where the greatest impacts on the species are likely to be across the area considered.

Using the USA as a model region, we investigated separately: the future distribution of climate change, sea-level rise, biological invasions, and habitat loss changes through the assessment of six land-use classes: *Developed, Mining, Disturbed forested lands*, *Grassland*, *Cropland*, and *Pasture*. We then determined the cumulative impact of these four main drivers of biodiversity loss for all the endemic species in the USA across four taxonomic groups (amphibians, birds, mammals, and reptiles). This novel metric identifies the overlap between spatially explicit species vulnerability and threats and identifies where potential impacts are greatest. Specifically, we attempted to answer the following questions: (i) Which are the areas most and least impacted by future threats? (ii) What is the relative contribution of different threats to the cumulative impact? (iii) Where and to what extent is USA endemic biodiversity (species and phylogenetic diversity) most vulnerable to these cumulative threats?

## Results

### Individual threats

**Figure 1:**
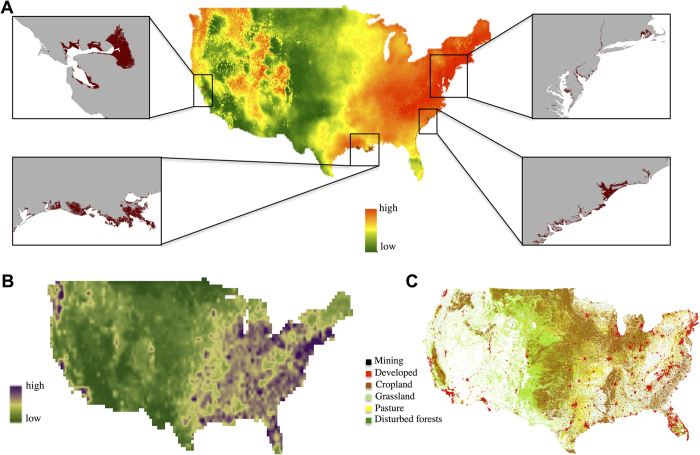
Threat maps for the 2080 s (A1B emission scenario) of (**A**) local climate change (mean of the three GCMs) and areas inundated by a 2 m increase in sea level, in highlighted squares and in brown; (**B**) predicted number of invasive alien species (mean of the three GCMs); (**C**) land-use classes including mining, developed, croplands, grasslands, pastures, and forested lands. Figure created with ArcGIS 10.2.1 software.

Only 0.31% of the surface area of the USA is predicted to be at risk of permanent inundation following a sea-level rise of 1 m. However, these predicted losses can be locally significant because they are concentrated in restricted coastal areas. Our results showed that sea-level rise will occur mainly in the states of Rhode Island (15%) and Delaware (7%). By contrast, local climate change (modification of temperature and precipitation) will be important and widespread over the eastern part and restricted to specific geographic areas across the western part of the USA under the A1B emission scenario (Fig. 1A). The predicted threats by invasive alien species and land-use showed a similar pattern, with a large area at risk throughout the eastern part and some restricted areas of high risk along the western coast (Fig. 1B,C).

### Cumulative threats

**Figure 2:**
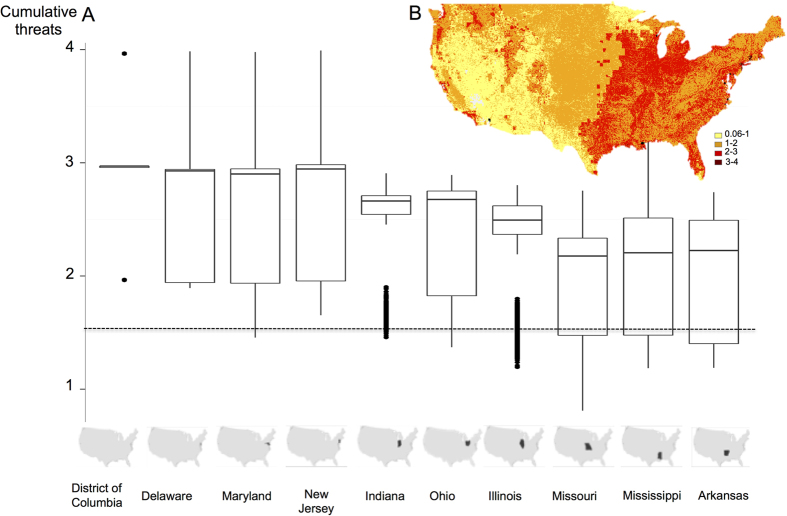
Cumulative threats in the 2080 s under the A1B emission scenario: (**A**) Boxplot of the ten most affected states in the USA under the A1B emission scenario. The horizontal grey line represents the median value of cumulative threats across the USA. (**B**) Spatial distribution figure created in R 3.1.1.

We also investigated the cumulative threats (that is, the total threat intensity values) occurring across the USA. On average, a minimum of 1.5 cumulative threat values per pixel occurred for the whole country. We observed substantial heterogeneity throughout the country, with a clear pattern of high cumulative threat values in the eastern part; low cumulative threat values are prevalent across most of the western part of the country under the two emission scenarios. Areas in the eastern half of the USA will be exposed to a minimum of two different threats simultaneously; the central part of the USA was predicted to be exposed to moderate cumulative threat values <2). In addition, the western part was predicted to be relatively less threatened except for the western coastline, which was exposed to high cumulative threat values (<4) (Fig. 2A; Figure S2). This trend becomes evident when summarizing the cumulative threat by state (Fig. 2 Table S3). The District of Columbia and Delaware were the areas most exposed to cumulative threat values under the A1B and B2A emission scenarios (median ≈3, Table S3). Maryland and New Jersey were also predicted to be affected by high cumulative threat values under the A1B emission scenarios but by lower values under the B2A emission scenarios. We also investigated exposure of species to cumulative threat values. Species at higher risk of extinction (and likely to have a smaller range size according to our data) were exposed to lower values of cumulative threats compared to species at low risk of extinction (with larger range size, except for “critically endangered” classified species, Figure S3). One of the explanation of this result might be due to the lower probability to be exposed to a high number of threat when the species range size is small. Moreover, the species that are at high risk of extinction were mainly located in the western part of U.S., where we found lower values of cumulative threats. In addition, related species of amphibians and mammals were generally exposed to similar cumulative threat values, because the phylogenetic signal was strong for these two groups (Supplementary Material 1).

### Cumulative impacts

**Figure 3:**
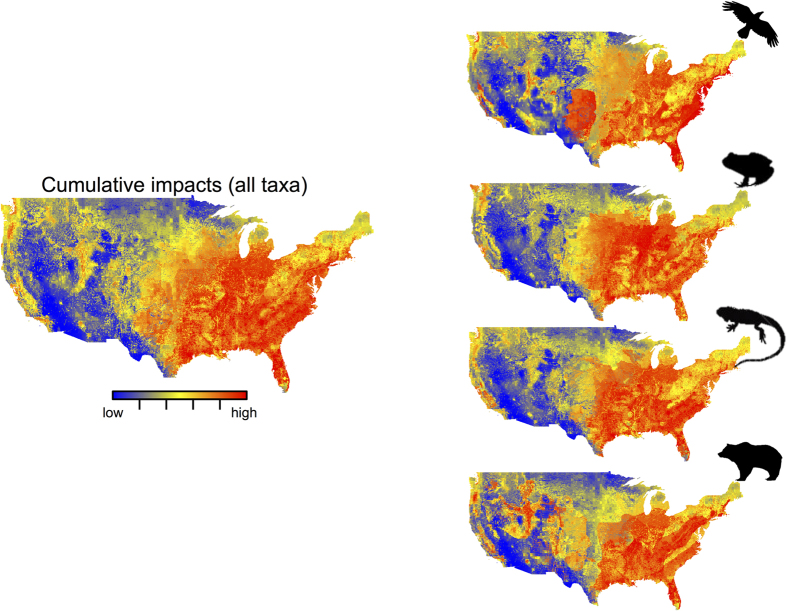
Cumulative impact maps for all the different taxa and for amphibians, reptiles, birds, and mammals in the 2080s for the A1B emission scenarios. The arrow represents cumulative impact values that go from low (blue) to high (red) values. Figure created in ArcGIS 10.2.1 software.

In addition to the cumulative threats, our cumulative impact index (that is, threats weighted by species vulnerability, and species distribution per pixel) also highlighted the same longitudinal gradient across the USA (Fig. 3). Large areas of moderate to high cumulative impact scores were also concentrated in the eastern part of the USA, while half of the western areas exhibited relatively low impact values (except for the western coastlines). This pattern was consistent across CO_2_ emission scenarios (B2A and A1B), time horizons (2050s and 2080s) and diversity metrics (phylogenetic and specific) (Figure S4-S5). We also found that the cumulative threat and cumulative impact value patterns differed at a finer scale. Of the states with the five highest cumulative threats, three were also among the states with the five highest cumulative impact values (i.e., except Illinois and Indiana), revealing the need to consider the spatial distribution of both the threats and the species vulnerable to these threats. The spatial variation of cumulative impact values also varied according to the taxa considered. For example, the cumulative impact values were very high for birds on the eastern coast of the USA while the values were lower for amphibians, mammals, and reptiles. High cumulative impact values were also apparent on the western coast and central areas for mammals: the other taxa were predicted to be less impacted there (Fig. 3). Moreover, while we predicted that cumulative impact values for birds, and mammals would be high in Texas, reptiles and amphibians were not predicted to be affected in this area. In addition, we found areas of high cumulative impact values in the eastern part of the USA for mammals (*e.g*., Idaho and Utah states).

### Influence of global changes and endemic species richness layers

**Figure 4:**
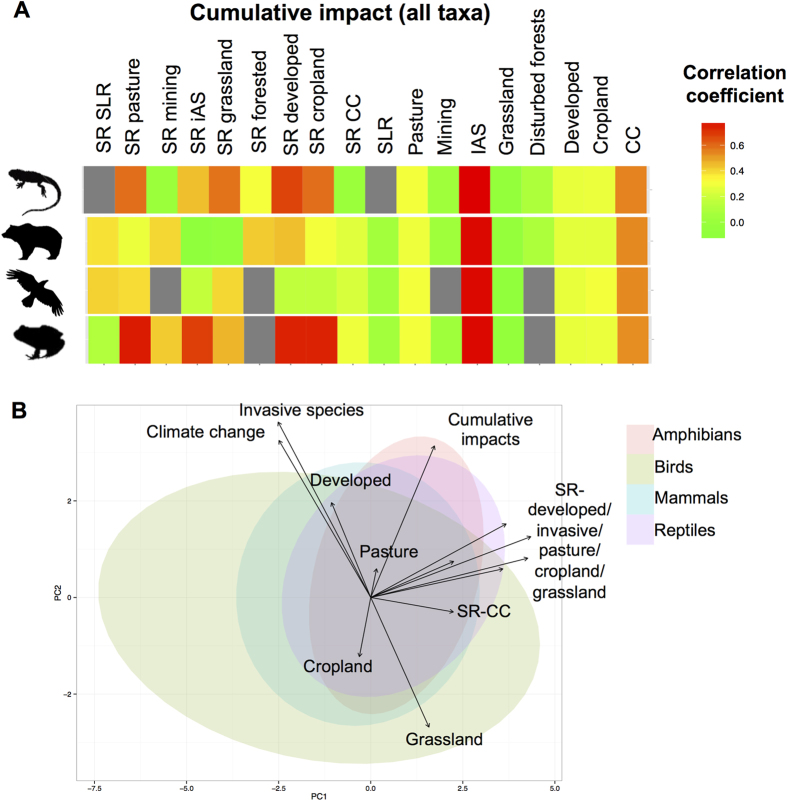
Relationship between cumulative impact values (in the 2080s under the A1B emission scenario) and individual threat intensities or species richness (SR) across the USA: (**A**) Correlation coefficient (Pearson) for each individual threat value with the cumulative impact values; (**B**) the PCA biplot showing the factors explaining the spatial variation of cumulative impact values and scores, colored by taxa. Acronyms: IAS (Invasive Alien Species), CC (Climate Change), SLR (Sea Level Rise), SR (Species Richness threatened by the given threat). Figure created in R 3.1.1.

The spatial distribution of threats and relative vulnerability of biodiversity to these threats play a major role in producing the observed spatial variation in cumulative impact values (Fig. 4). Invasive alien species followed by climate change, and change of land use to croplands and pastures showed broad positive correlation with cumulative impact values across the different taxa (Fig. 4A). In addition, endemic species richness vulnerable to invasive alien species, *Developed*, *Cropland*, *Grassland,* and *Pasture* were also positively correlated with cumulative impact values. Hence, variation in cumulative impacts was driven largely by concordant spatial patterns in multiple threats (*i.e.,* invasive species, climate change, *Developed, Cropland*, *Grassland*, and *Pasture* areas) and high species richness vulnerable to these threats (Fig. 4B). More specifically, some threats were associated with high cumulative impact values on some taxa (see also Figure S6, 7, 8, and 9). For example, invasive alien species, *Cropland*, *Developed* and the presence of amphibians vulnerable to these threats were positively correlated with high cumulative impact values (Fig. 4A and S6). Species vulnerable to Developed, *Grassland, Pasture*, and *Cropland* threats also showed a broad positive correlation with cumulative impact values for reptiles; other taxa such as mammals were also predicted to be particularly vulnerable to mining areas (Fig. 4A).

Overall, sensitivity analyses demonstrated that spatial patterns of cumulative impacts were robust to alternative normalization methods (Pearson’s r: 0.810), date (Pearson’s r: 0.977) or CO_2_ emission scenarios (Pearson’s r: 0.894) (SI 3 for details about sensitivity tests of standardization methods and outputs).

## Discussion

This is the first attempt to create a spatially explicit assessment of future cumulative threats to terrestrial biodiversity, including their specific impacts on terrestrial biodiversity at a large scale (but see^9^ for cumulative threat values). We combined four main drivers of biodiversity loss, weighted by presence of endemic species vulnerable to these threats across the USA. We showed that in general regions that are likely to be exposed to these futures threats coincide with the localization of endemic species vulnerable to these threats.

We found that local climate change, invasive alien species, and land-use classes such as *Cropland*, *Pasture*, and *Developed* areas were aggregated over the eastern part of the USA; climate change was the main factor in the western part of the USA. These results were in accordance with previous single-threat studies showing the effects of climate change^3^ and land-use classes^10^ would occur mainly in the eastern part of the USA. Overall, our findings demonstrated high cumulative threat values (>2 threats) throughout the eastern part of the country, with lower values in the central and western parts. This result is particularly important because the combined impacts of these threats should be larger than the cumulative effect of each threat on biodiversity^2^. Indeed, we can expect synergistic effects among climate change, land-use patterns and invasive alien species on biodiversity throughout the eastern part of the country because these multiple threats are likely to occur simultaneously. However, because little is known about the possible existence of interacting effects, quantifying the magnitude of potential synergies should become a priority. Moreover, this result also reveals that studying future threats in isolation will lead to a partial interpretation of the global spatial distribution and impact of threats, particularly in the eastern half of the country. Studying spatial distribution of threats individually is clearly necessary to determine appropriate conservation measures. However, multiple threats operate simultaneously, generating cumulative impacts. Major investments in remediating a subset of threats at a particular location may have little net benefit if other threats remain unaddressed^5^. Therefore, our analyses, combined with single threat analyses at the finest resolution, will allow targeted investment in optimal strategies for a wide range of threats that differ in relative impact on different taxonomic groups (depending on the vulnerability of species to these threats). The cumulative impact index will therefore allow identification of areas that are relatively more exposed to cumulative impacts and of high interest for conservation (high number of vulnerable species).

All the studies that consider the spatial distribution of future threats relative to the implementation of conservation measures have disregarded the vulnerability of species to these threats (but see Ref. 11). Our cumulative impact values showed some spatial disparities with the cumulative threat values, particularly across the central and northeastern regions, thus revealing the importance of accounting for species vulnerability to these threats. Overall, nearly half of the eastern part of the USA showed high cumulative impact values. This result highlights that the distribution and magnitude of threats and species vulnerabilities to these threats contribute to cumulative impact patterns that cannot be anticipated from threat analyses alone. We considered only endemic species richness, to ensure that weighted values were associated with threats actually occurring in the USA. In addition, we explicitly considered the spatial distribution of species vulnerable to each threat to avoid over or under-estimation of their vulnerability to these threats. Although species richness (not only from endemic species) using IUCN (International Union for Conservation of Nature) data revealed the same pattern across the USA (Figure S10), the vulnerability weights associated with taxa may change in a non-congruent way. Therefore, our results regarding cumulative impacts are not transferable to species richness across the US. However, our results were robust to the metrics of diversity used (either species or phylogenetic diversity), which is consistent with the positive and monotonic relationship between phylogenetic diversity and species richness of species assemblages^12^.

We predicted that biodiversity in the USA will be threatened mainly by invasive alien species, followed by climate change, and the development of *cropland, urban* and *pasture* areas in the future. Moreover, amphibian and reptiles species vulnerable to land use changes were particularly exposed to these threats, while other taxonomic groups were predicted to be less affected. This result is in agreement with Riordan *et al.* (2014), who predicted that land-use changes would result in higher losses for many species^13^ when compared with climate change. This result is partly because fewer mammals (21%) and amphibian species (15%) are currently threatened by climate change, according to the IUCN threat classification scheme. Naturally, using the IUCN threat classification scheme to calculate the “vulnerability weight” assumes that the current responses of species to threats are representative and accurate for future threats within the USA, which is not necessarily the case. This shortcoming can lead to underestimation of the future impacts of climate change (for mammals and amphibians), pollution (for amphibians), and overexploitation (for amphibians, birds, mammals, and reptiles) (Table S4). Indeed, a significant proportion of USA endemic amphibians, mammals, birds, and reptiles are endemic to the USA are significantly threatened by overexploitation, and this is likely to continue into the future. In this sense, our results are conservative. However, this approach is one of the most comprehensive ways of including vulnerability of species to future threats. Another possibility would be to take into account life history traits of species that are likely to be associated with vulnerability to future threats^11^,^14^,^15^,^16^,^17^ but this would require a significant amount of information that is not yet available. In addition, although the development of urban areas currently represents the main threat to species across the USA (Table S4), this threat was not predicted to have an important impact in the future.

Because cumulative impact analyses have some limitations, the results should be interpreted with caution. First, different threats operate at different scales^16^, so the reliability of the global results for local or regional use is still unclear (see also Ref. 6). Although we studied the main threats that are likely to impact biodiversity in the future, it is likely that some unidentified threats may be locally important^17^, including overexploitation, pollution (i.e., amphibians) or emergent diseases. For example, overexploitation is one of the most important current threats for amphibians (19%), birds (12%), mammals (15%), and reptiles (11%) (Table S4). However, lack of data on these future threats prevented us from including them in this analysis. In addition, the spatial resolution of the threat variables might have influenced our results. In particular, the climate change results could have been overestimated because we did not consider the microclimate conditions that might occur at the finest resolution and serve as refuges for some species. In addition, our cumulative impact values do not capture the dynamic processes of species responses. Indeed, we assumed that the species would respond linearly to increases in the intensity of the threat. We also assumed that species vulnerability to these threats is likely to remain the same in the future. Future steps should include close monitoring of biodiversity to assess whether species vulnerability to these threats changes over time. For example, we would expect greater vulnerability of many species to climate change in the future (see Ref. 11,15). Due to data availability, we examined a subset of four taxa, but similar studies of other taxonomic groups such as invertebrates and plants, would also be important. Similarly, other geographical regions could be studied using this method.

Although our results should be considered with caution they have significant implications for conservation measures. To design and implement effective conservation measures, we need to understand the potential cumulative impact of the full suite of threats on multiple taxa^18^. The need for cumulative impact assessments for conservation purposes has grown recently, particularly in the USA (e.g., the National Environmental Policy Act, (http://www.epa.gov/compliance/resources/policies/nepa/cumulative.pdf). The cost-effectiveness of investments is likely to be increased when conservation actions take into account the impacts of multiple threats^19^. For example, common conservation measures could be implemented to protect biodiversity from both climate and land-use changes (e.g., the development of protected areas and connectivity between these protected areas). Cumulative impact analyses also suggest that the return on investments for conservation purposes may be low when high cumulative threat areas with low species diversity require protection from many threats (e.g., Minnesota, Wisconsin, Michigan, Iowa states). By contrast, areas with moderate cumulative threat values and a high diversity of species vulnerable to these threats might be of great concern for conservation purposes (e.g., Oklahoma, Texas, and Kansas). These states have relatively high cumulative impact values despite moderate values of cumulative threats (Figs 2,3). Therefore, these states would not be identified as high priorities based on an analysis of threats alone (e.g., climate change or invasive species), although a high number of species might be threatened.

In conclusion, our results highlight the importance of taking both threats and species vulnerability to these threats into account to define the impact of global changes on biodiversity. We also show the need for increasing research on cumulative impacts to aid assessments of the effects of future global changes: this knowledge will also improve implementation of conservation measures by incorporating complex multiple threat-taxa combinations. This approach should be applied to future threat impact assessment in other regions of the world, which would support development of strategies for simultaneously mitigating impacts and managing species.

## Methods

### Threat layers

We decided to focus on habitat loss, which is the most important driver of the current loss of biodiversity at a global scale^20^,^21^,^22^,^23^, followed by biological invasions^24^,^25^. In addition, we also considered climate change, which is likely to become the most important driver of future of biodiversity^26^. Because of their relative importance for explaining the current pattern of threatened species at a global scale and of data availability for future scenarios, we focused our analyses of future threats on the following issues: climate change, sea-level rise, invasive species, and habitat loss (Table S4). In addition, the intensity of climate change, biological invasions and habitat losses are likely to change in the future. These threats will also act simultaneously and synergistically in the future^28^,^29^,^30^,^31^, justifying the decision to combine them in one analysis. Unfortunately, it was not possible to include other major threats to biodiversity, such as pollution, nitrogen deposition or overexploitation, either because no forecast for these threats exists at a global scale or because the IUCN category does not explicitly include a threat in its categories. Therefore, it would have not been possible to calculate the proportion of endemic species vulnerable to the threat per pixel.

#### Sea-level rise

We determined the land area that will be permanently submerged by 1 m and 2 m sea-level rises, using the same methodology as in Ref. 32. We extracted elevation data with 250 m resolution for the United States from the digital elevation model developed by NASAs shuttle radar topography mission^33^. Potentially submerged areas include standing freshwater such as rivers, wetlands, and associated habitats connected to the ocean. We only considered flooded cells that connected to the ocean below a projected sea-level rise. This data layer populates each cell with a binary value of presence/absence of submersion by sea-level-rise scenario. We considered two sea-level-rise scenarios: 1 and 2 m.

#### Climate change

We calculated the local climate change defined as the standardized Euclidean distance (SED) between the current and future climate data for each pixel (see formula below).
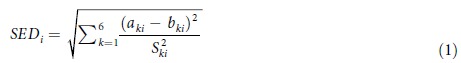
where *a*_*ki*_ and *b*_*ki*_ are the current and future means for climate variable *k* at grid point *i,* and *S*_*ki*_ is the standardized current climate value, following the established methodology^34^. We used six different climate variables linked to temperature and precipitation (i.e., annual mean temperature, maximum temperature of warmest month, minimum temperature of coldest month, annual precipitation, precipitation of wettest month and precipitation of driest month). Current climatic data were averaged from the 1950–2000 data of the WorldClim database^35^,^36^, and future climate data were obtained from the Global Climate Model data portal for the 2050s and 2080s (Global Climate Model, 2013); all data had a resolution of 10′ resolution (*i.e.,* 18.6 × 18.6 km). Simulations of future climates were based on three general circulation models (i.e., HADCM3, CSIRO2 and CGCM2) and two CO_2_ emission scenarios (i.e., B2A and A1B). This data layer assigns continuous values of an index of local climate change to each pixel.

#### Invasive alien species

We used the projected threat of the 100 among the world’s worst invasive alien species modeled by Ref. 29 for the 2050s and 2080s under the A1B and B2A emissions scenarios at 0.5° resolution. The IUCN list of “*100 of the world’s worst invasive alien species*” provides the most geographically and taxonomically representative set of the most dangerous invasive alien species around the world; the species significantly affect biodiversity and/or human activity in all ecosystems^37^. From the list, we kept 57 invasive alien species known to be present in the United States: ten terrestrial invertebrates, seven mammals, seven freshwater fishes, five aquatic invertebrates, four fungi, three amphibians, one aquatic plant, one bird, and one reptile, and 18 terrestrial plants. This data layer assigns a continuous value of the number of invasive alien species to each pixel.

#### Land-use change

We used projected land-use and land-cover scenarios for the 2050s and 2080s under two emissions scenarios (i.e., B2A and A1B) developed by the United States Geological Survey using the FORecasting SCEnarios of future land-cover (FORE-SCE) model at 250 m resolution^38^. These spatially explicit models integrate both “topdown” drivers of land cover/land-use, such as demographic change, and local-scale “bottom-up” drivers, such as biophysical site conditions^39^. The USGS land-use/land-cover maps encompass 17 classes, but we considered only the six classes corresponding to the same classes in the IUCN classification scheme (*i.e., Developed, Mining, Disturbed forested lands*, *Grassland*, *Cropland* and *Pasture*). These six classes of threat are represented by binary values of presence/absence for each pixel.

Because the native resolution of the variables ranges from 250 m to 0.5 degrees, we had to aggregate the cumulative impact index at the coarser resolution. We first preserved the quantitative information for sea-level rise and land use at the 250 m resolution to avoid underestimation of these threats. Then, we aggregated the cumulative threat index to the same resolution. Ideally, all the threat layers should have had a resolution of 250 m. However, climate and invasive alien species layers do not exist at this resolution at the global scale. Consequently, we aggregated all the data to the lower common denominator, which ensured that all the data could be accurately presented at the same resolution; therefore valuable information about finer-scale spatial distribution of stressors (i.e., land use and sea-level rise) was discarded^8^. We also assumed an uniform distribution of threats within a pixel, which is unlikely to occur. This assumption is particularly relevant for threats with coarser native resolutions, such as climate change and invasive species.

### The cumulative impact approach follows a 3-step process

First, we assembled the layer for each threat. Second, we transformed and rescaled each threat from 0 to 1 to create a single unitless scale. Third, for each pixel, we multiplied each threat by the species richness per taxa to create threat-by-taxa combinations and then multiplied these combinations by the appropriate weighting value that is spatially explicit. This weighting value represents the relative number of species that are vulnerable to a threat for a given pixel using IUCN data (*i.e.,* cumulative impact index; Figure S1 for a summary of the methodology used).

### Cumulative impact index

We adapted the cumulative impact index (CI) developed by Ref. 6 to combine multiple future threat categories into a single comparable estimate of cumulative impact on biodiversity for four taxonomic groups. Our cumulative impact index was defined as the normalized value of the intensity per threat multiplied by normalized species richness per taxa and by the relative weight of each threat-taxa combination, pixel by pixel.
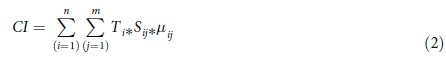


Where *T*_*i*_ is the normalized value of the intensity of a threat (scaled from 0 to 1) at location *i*, *Si*_*j*_ is the normalized value of species richness per taxa *j at location i* (from 0 to 1), and *μ*_*ij*_ is the weight of a threat at location *i* per taxa *j* (range 0 to 1). Here, we used *n* = 9 and *m* = 4 taxa (see below).

#### Future threats category (T)

We used data from four main drivers that are likely to affect the future biodiversity of the USA. These threats included climate change, sea-level rise, invasive species, land-use changes (using six land-use classes: *Developed*, *Mining*, *Disturbed Forested lands*, *Grassland*, *Cropland*, and *Pasture* (see SI text for details)). All these threats were generated for the 2050s and 2080s under two CO_2_ emission scenarios (except for sea-level rise, for which we considered two scenarios of 1 and 2 meters).

#### Endemic species data (S)

We used occurrences of endemic species of amphibians, birds, mammals, and reptiles in the USA, provided by the IUCN and Birdlife. To be sure that these threats are currently threatening the species within the USA, we chose to keep only endemic species. In accordance with the threats classification scheme defined by the IUCN (version 3.2), we selected 196 species affected by invasive species, climate change, sea-level rise, developed land use, mining, mechanically disturbed forested land use, grassland, cropland, and pasture land (Table S2 for detailed IUCN classification). We created species richness maps at 10′ by using data from the IUCN Red List (November 2013 version) that were resampled at 250 m resolution for the sea-level-rise threat. These data layers assign a continuous value of endemic species richness to each pixel. We assumed that current endemic species richness will be unchanged in the future because (i) the species are endemics and are therefore likely to maintain their distribution through time unless major threats occur and (ii) modeling the potential distribution of the endemic species in the future would increase the uncertainties in our analyses.

#### Phylogenetic signal in species’ exposure

The phylogenetic distance between species was directly calculated from phylogenetic trees. We used a complete phylogeny for birds^40^, amphibians^41^, reptiles^42^ and mammals^43^. Phylogenetic diversity was calculated as the “sum of the lengths of all the branches that are members of the corresponding minimum spanning path”^44^,^45^, in which “branch” is a segment of a tree, and the minimum spanning path is the minimum patristic distance between the two nodes. To estimate whether there was a phylogenetic signal in species’ exposure, we used the maximum-likelihood based measurement of phylogenetic signal, namely the lambda model, as developed by Pagel^46^. This metric corresponds to a tree transformation parameter that gradually eliminates phylogenetic structure when varying from 1 to 0. Lambda values of 1 correspond to a Brownian evolution, whereas a lambda value of 0 corresponds to the complete absence of phylogenetic structure.

#### Data standardization

We rescaled the pixel values of each individual threat, species richness, and cumulative impact values to a 0–1 range to create a unitless metric of the intensity of a given threat 5,6. Following^5^, we tested three types of transformations to accomplish the 0–1 standardization per threat and species richness value. First, we used a natural-log transformation (ln[x + 1]) before max-min rescaling, thereby compressing the upper end and expanding the lower end of the range. This process minimizes the influence of extremely high values that could be spurious yet influential and retains all quantitative spatial information. The majority of studies of ecological impact of stressors use this method^6^. Second we used a basic standardization, a max-min linear rescaling ([x_i _– x_min_]/[x_max_ – x_min_]). Third we used a cumulative distribution function to replace each value with a score reflecting its percentile rank relative to all other pixels^5^. The cumulative distribution function approach resulted in a uniform number of pixels at any given level of value, whereas the linear and log transformations allowed heterogeneity in the number of pixels at any given level of value.

#### Weights according to threat and species (μ)

In previous studies, one key innovation of the cumulative impact mapping process was the development of unique and quantitative vulnerability weights for a given threat. This innovation was mainly relying on expert opinions^6^,^47^,^48^, which has been criticized because of potential biases in expert judgment, see^8^. In this context, Halpern *et al.*^8^ advocate to use very large numbers of experts to account for these potential biases and then assume those responses are representative and accurate across space. In this sense, the IUCN database is a useful tool because the IUCN supervises a process by which major threats affecting species are spatially identified and classified^49^ by a very large number of experts worldwide. Moreover, we decided to define the weights of each threat according to the proportion of species currently threatened by these threats for each pixel across USA.

#### PCA analyses

We also performed principal components analysis (PCA) using the prcomp function in R to assess whether combinations of threats or species richness vulnerable to these threats could summarize most of the variation among high stress pixels^5^. We found that the two first components explained 44.60% of the variation in cumulative impacts.

### Sensitivity tests of calculation methods

Our analysis required some decisions about standardization approaches that could have influenced our conclusions. Therefore, we tested the sensitivity of our results to the standardization procedure by calculating the spatial correlation between the different maps of cumulative impact values on 250,000 randomly extracted pixels for computing reasons.

All the analyses were performed for the 48 mainland states of the USA: as we excluded Hawaii and Alaska from the analyses.

## Additional Information

**How to cite this article**: Bellard, C. *et al.* Combined impacts of global changes on biodiversity across the USA. *Sci. Rep.* **5**, 11828; doi: 10.1038/srep11828 (2015).

## Supplementary Material

Supplementary Information
