# Supplementary material for: Combined impacts of global changes on biodiversity across the USA
Source: Sci Rep. 2015 Jul 7;5:11828. doi: 10.1038/srep11828 (PMC4493580; doi:10.1038/srep11828)
Supplement: Supplementary Information [file srep11828-s1.pdf]

Supplementary material information:

Combined impacts of global changes on biodiversity across the USA

C. Bellard, C. Leclerc, & F. Courchamp

Table S1. Sources and characterization of spatial data used in mapping U.S. future threats. All the layers used the same WGS84 projection).

Threat	Native data type and scale/cell size	Threat value	Scaling method	Data sources
**Sea level rise**	Raster ; 250m resolution (WGS84 projection)	Binary threat	--	Jarvis et al. 2008
**Climate change**	Raster; 10 arc minutes (WGS84 projection)	Continuous threat	Resampled on sea level rise map	Hijmans et al. 2005
**Invasive species**	Raster; 0.5° resolution (WGS84 projection)	Continuous threat	Resampled on sea level rise map	Bellard et al., (2013)
**Land-use change**	Raster; 250m (Albers projection)	Binary threat	Resampled on sea level rise map	Sleeter et al. 2012

Table S2. Number of endemic species per taxa categorized as ongoing threatened by different drivers. We only kept species considered as Data Deficient, Least Concerned, Near Threatened, Vulnerable, Endangered and Critically endangered species. The number in grey represents the threats classification scheme (Version 3.2) of IUCN that have been considered in this study.

In order to measure the potential vulnerability of species to future threats, we used the IUCN classification. This classification allows us to establish the number of species evaluated by the IUCN as already at risk of extinction due to a given threat. In this aim, we only used endemic species to USA to be sure that the given threat occurred in USA and not in another country. We also removed from the analyses species that are already extinct because they are not present in the US.
First, using the IUCN spatial data and Birdlife spatial data, we extracted the polygons of mammals, amphibians, reptiles and birds species that were only in USA. Then, we calculated the number of species within each category of threat that we have selected as relevant for the threat of interest (see IUCN category in grey for details). For example, we selected species that are classified as threatened due to *Storms & Flooding* following the IUCN category to calculate the number of species vulnerable to sea-level rise (N=9). Finally, we used the number of species threatened per category to estimate the weights attributed for each threat layer by taxa calculated from IUCN data (See Table S3).

	Taxa			
Threat *(IUCN category)*	***Amphibians***	***Birds***	***Mammals***	***Reptiles***
Sea level rise 11.4	4	2	3	-
Climate change 11.1, 11.5	16	7	9	2
Invasive species 5.1.2, 8.1.1, 8.1.2, 8.4.1, 8.4.2	29	4	21	6
Developed 1.1 to 1.3, 4.1, 4.2, 4.4, 7.2.1, 7.2.2, 7.2.5, 7.2.6, 9.1.1 to 9.1.3, 9.2.1 to 9.2.3, 9.6.1 to 9.6.4	78	5	30	26
Mining 3.2	16	-	6	7
Forested lands 5.2.2, 5.2.3	-	-	1	1
Grassland 2.3.1 to 2.3.4, 9.3.2	24	3	19	9
Cropland 2.1.1 to 2.1.4, 2.2.1 to	51	6	28	24

2.2.3, 7.2.3, 7.2.7, 9.3.1 to 9.3.4				
Pasture 2.1.1 to 2.1.4, 2.2.1 to 2.2.3, 2.3.1 to 2.3.4, 7.2.3, 7.2.7, 9.3.1 to 9.3.4	62	7	31	27

Table S3. Cumulative threats values for U.S. states under the two emission scenarios (A1B, B2A) and timeframe (2050s, 2080s).

Within the 10 top ranked states that are exposed to the highest cumulative threat values (under the A1B emission scenario in 2080s, see in bold in the Table), the majority of these states (>6) are also in the 10 top ranked exposed states under the B2A scenario. For the other four states that are ranked among the 10 most exposed states to high cumulative threats values under the A1B emission scenarios, they are respectively ranked at the 11, 12, 22 and 23 position under the B2A emission scenarios in 2080s.

	Cumulative threat values							
	**A1B 2050**		**B2A 2050**		**A1B 2080**		**B2A 2080**	
**States**	*median*	*sd*	*median*	*sd*	*median*	*sd*	*median*	*sd*
Alabama	1.68	0.50	1.69	0.47	1.71	0.51	1.80	0.46
Arizona	0.70	0.44	0.75	0.43	0.64	0.44	0.56	0.44
**Arkansas**	1.61	0.54	1.77	0.55	2.22	0.54	1.72	0.53
California	0.88	0.64	0.99	0.67	0.87	0.64	1.00	0.69
Colorado	1.23	0.41	1.28	0.46	1.22	0.41	1.27	0.47
Connecticut	1.98	0.52	1.91	0.52	1.99	0.53	1.96	0.51
**Delaware**	2.84	0.49	2.70	0.56	2.93	0.48	2.74	0.51
**District of Columbia**	2.97	0.23	2.83	0.24	2.97	0.23	2.84	0.24
Florida	1.66	0.52	1.53	0.52	1.99	0.54	1.59	0.54
Georgia	1.76	0.52	1.57	0.48	1.82	0.53	1.79	0.47
Idaho	1.13	0.53	0.96	0.53	1.15	0.53	0.95	0.53
**Illinois**	2.45	0.38	2.41	0.51	2.49	0.36	2.22	0.42
**Indiana**	2.63	0.39	2.50	0.53	2.66	0.37	2.32	0.43
Iowa	1.97	0.32	2.02	0.48	1.97	0.32	1.89	0.40
Kansas	1.62	0.32	1.94	0.54	1.61	0.32	1.94	0.54
Kentucky	1.79	0.54	1.70	0.55	1.85	0.54	1.67	0.51
Louisiana	1.70	0.56	1.91	0.50	2.08	0.59	1.83	0.49
Maine	1.50	0.38	1.49	0.34	1.50	0.41	1.49	0.35
**Maryland**	2.84	0.53	1.85	0.55	2.90	0.53	1.90	0.54
Massachusetts	1.96	0.53	1.91	0.51	1.98	0.58	1.96	0.51
Michigan	1.64	0.61	1.23	0.69	1.71	0.61	1.24	0.58

Minnesota	1.34	0.64	1.02	0.69	1.41	0.64	1.22	0.55
**Mississippi**	1.60	0.53	1.78	0.52	2.20	0.53	1.72	0.49
**Missouri**	2.16	0.49	2.31	0.57	2.18	0.48	2.18	0.53
Montana	1.29	0.43	1.18	0.49	1.35	0.42	1.22	0.49
Nebraska	1.55	0.32	1.49	0.49	1.56	0.32	1.54	0.46
Nevada	0.65	0.38	0.68	0.35	0.59	0.39	0.63	0.36
New Hampshire	1.59	0.42	1.60	0.38	1.60	0.45	1.61	0.38
**New Jersey**	2.69	0.52	1.87	0.52	2.95	0.53	1.93	0.51
New Mexico	1.05	0.51	1.02	0.51	1.02	0.51	0.97	0.53
New York	1.71	0.55	1.61	0.54	1.75	0.56	1.64	0.52
North Carolina	1.91	0.54	1.79	0.51	1.96	0.55	1.74	0.49
North Dakota	1.30	0.30	1.21	0.49	1.35	0.30	1.26	0.48
**Ohio**	2.64	0.48	2.51	0.57	2.68	0.47	2.38	0.49
Oklahoma	1.58	0.46	1.96	0.53	1.59	0.45	2.06	0.54
Oregon	0.97	0.58	0.95	0.57	0.93	0.60	0.99	0.56
Pennsylvania	1.85	0.55	1.64	0.56	1.88	0.55	1.66	0.54
Rhode Island	1.87	0.50	1.85	0.48	1.94	0.66	1.91	0.47
South Carolina	1.84	0.52	1.68	0.49	1.92	0.54	1.74	0.47
South Dakota	1.51	0.32	1.40	0.48	1.57	0.31	1.45	0.43
Tennessee	1.85	0.50	1.78	0.51	1.87	0.51	1.80	0.48
Texas	1.41	0.67	1.50	0.71	1.41	0.69	1.56	0.69
Utah	0.86	0.52	0.85	0.55	0.84	0.53	0.82	0.55
Vermont	1.57	0.45	1.51	0.42	1.61	0.45	1.52	0.41
Virginia	1.89	0.53	1.79	0.47	1.93	0.53	1.73	0.46
Washington	1.43	0.53	1.28	0.55	1.42	0.54	1.29	0.54
West Virginia	1.63	0.42	1.58	0.37	1.63	0.43	1.51	0.37
Wisconsin	1.81	0.60	0.92	0.66	1.92	0.60	1.08	0.55
Wyoming	1.11	0.58	1.01	0.60	1.17	0.57	1.02	0.61

Table S4. Percentage of endemic species in US threatened according to the IUCN classification. Only the three most important threats per taxonomic group have been represented here. The threats in grey represent the two threats that have not been considered in the current study because of data availability for the future timeframe.

Threat	Amphibians	Birds	Mammals	Reptiles
**Urban areas**	21%	-	18%	28%
**Agriculture**	-	-	25%	26%
Biological resource use	19%	12%	15%	11%
Pollution	15%	-	-	-
**Climate change**	-	13%	-	-
**Invasive**	-	22%	-	-

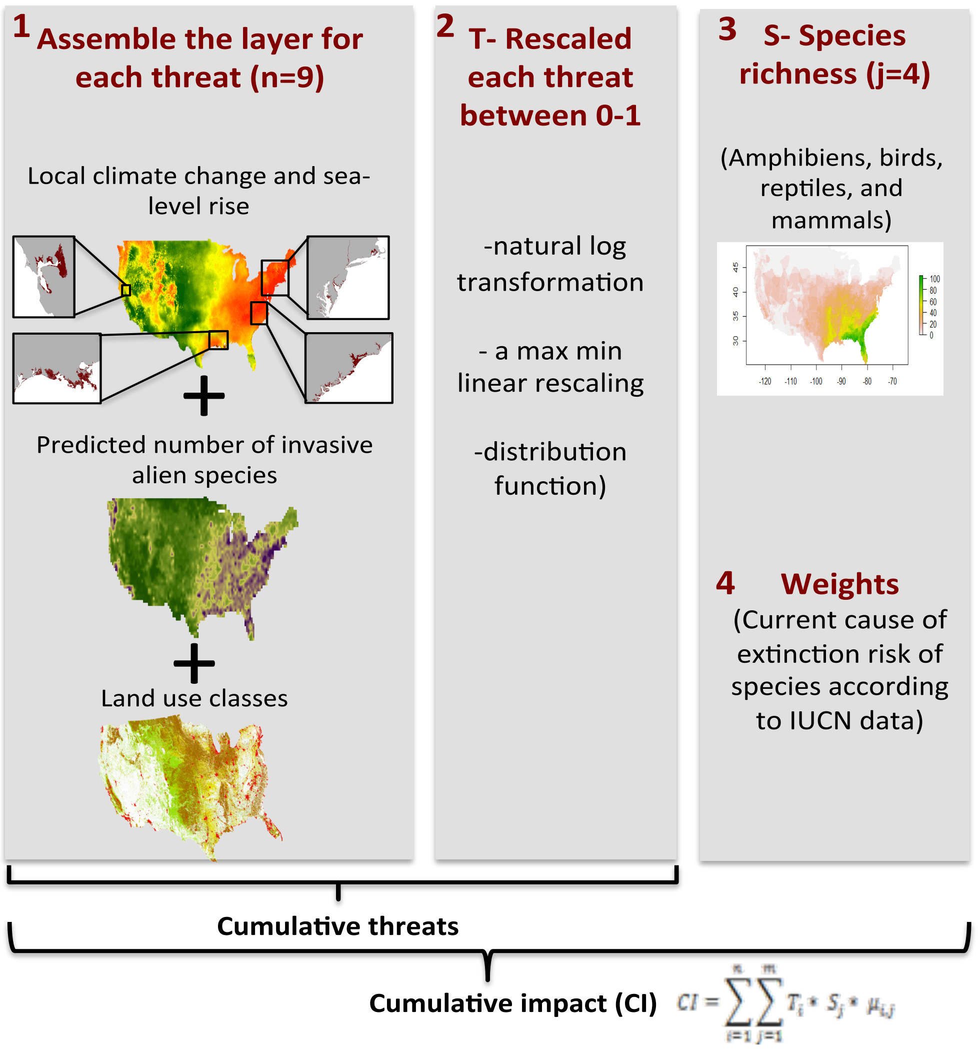

Figure S1. Summary of the methodology used to calculate the cumulative impact

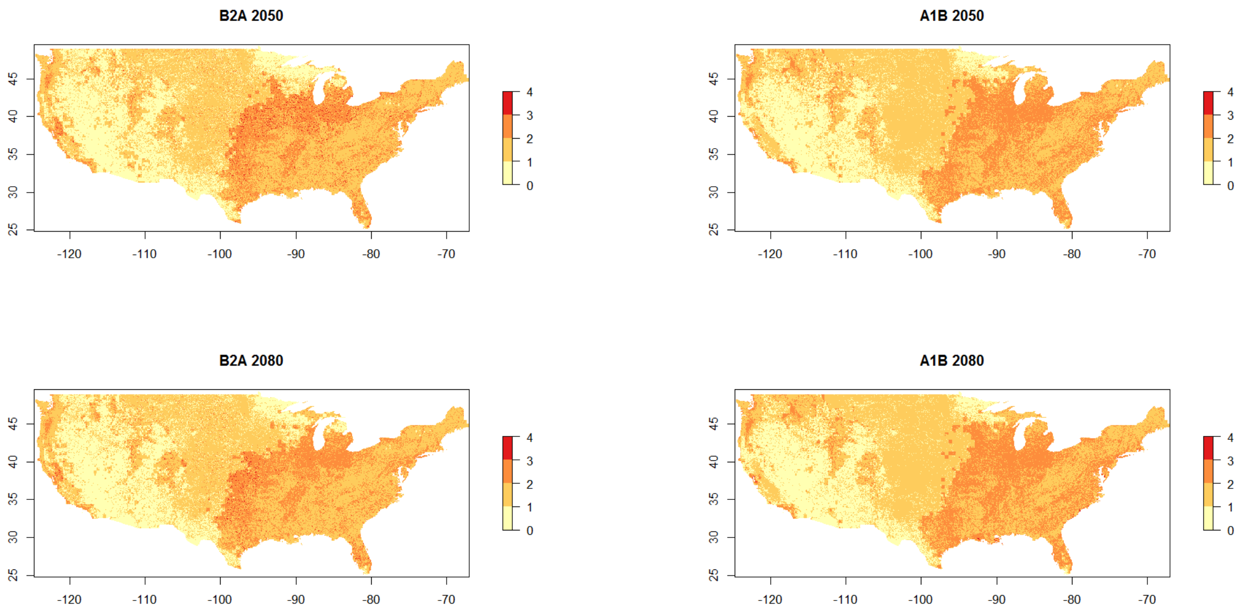

Figure S2: Cumulative threat maps for two emission scenarios (A1B and B2A) and two dates (2050-2080). Across the emission scenarios and dates we observed a similar pattern of high cumulative threat values. Indeed, most of the high cumulative threat values are located on the eastern part of the USA. On the contrary, most of the low cumulative threat values (<2) are located on the western part for the two emission scenarios and periods. We also observed that for most of the dates and scenarios a band of high cumulative threat values along the western coast of the United States. Therefore, we did not observe strong differences between the A1B and B2A scenarios. However, we can noticed that a significant part of the central states of the USA (Texas, Oklahoma, Kansas states) are predicted to be exposed to highest cumulative threat values under the B2A scenario compared to the A1B in 2080s. On the contrary, a larger part of the states that are located in the northwest of the US (Wisonsin, Minnesota, Iowa, Illinois) are predicted to be affected by high cumulative threat values under the A1B scenario compared to the B2A scenario. Figure created in ArcGIS 10.2.1 software.

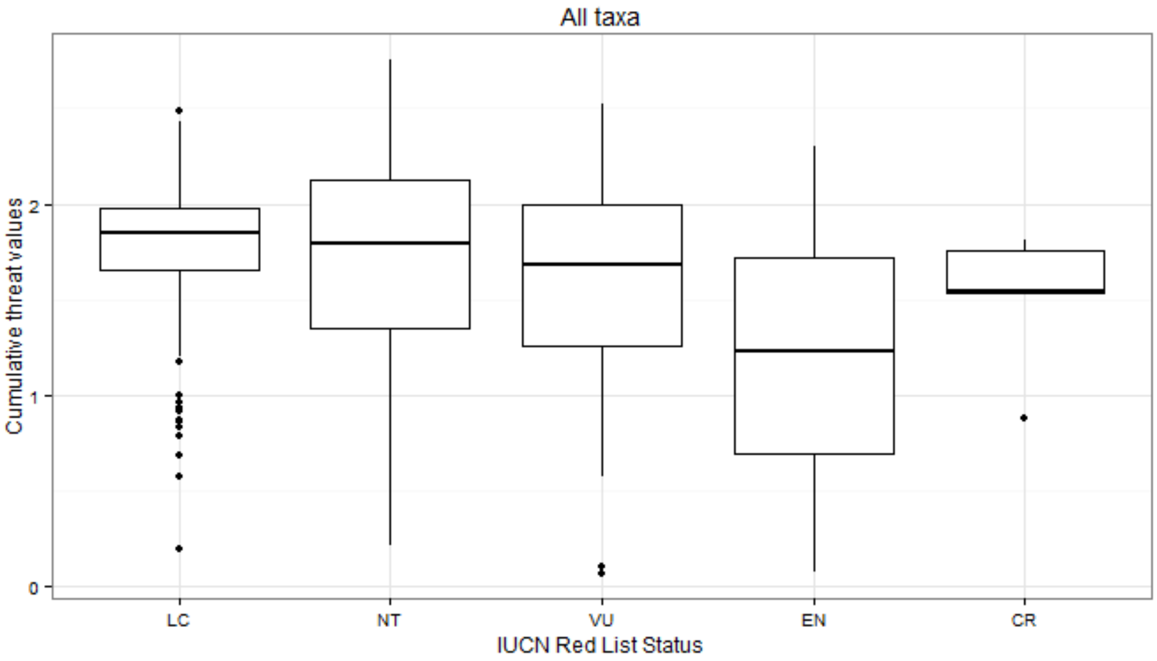

Figure S3. Cumulative threats values for the species according to their IUCN category status from LC ("Least concern"), NT ("Near Threatened"), VU ("Vulnerable"), EN ("Endangered"), and CR ("Critically Endangered") under A1B scenarios 2080.

For each species, we calculated the median of the cumulative threat values across their respective area distribution. Then, we did a boxplot of the cumulative threat values for all the species per IUCN category using the ggplot() package in R. We found that species at higher risk of extinction (that are likely to have a small range size) are less likely to be affected by high cumulative threat values (except for CR species) compared to species at lower risk of extinction (that are likely to have a large range size). One of the explanation of this result might be due to the species that are at high risk of extinction are mainly located in the western part of U.S. (where we calculated lower values of cumulative threat) or due to the range size of the species. Indeed, in our analyses species at low risk of extinction are likely to have a larger range. Therefore species might be more likely to be exposed to high values of cumulative threats. Figure created in R 3.1.1.

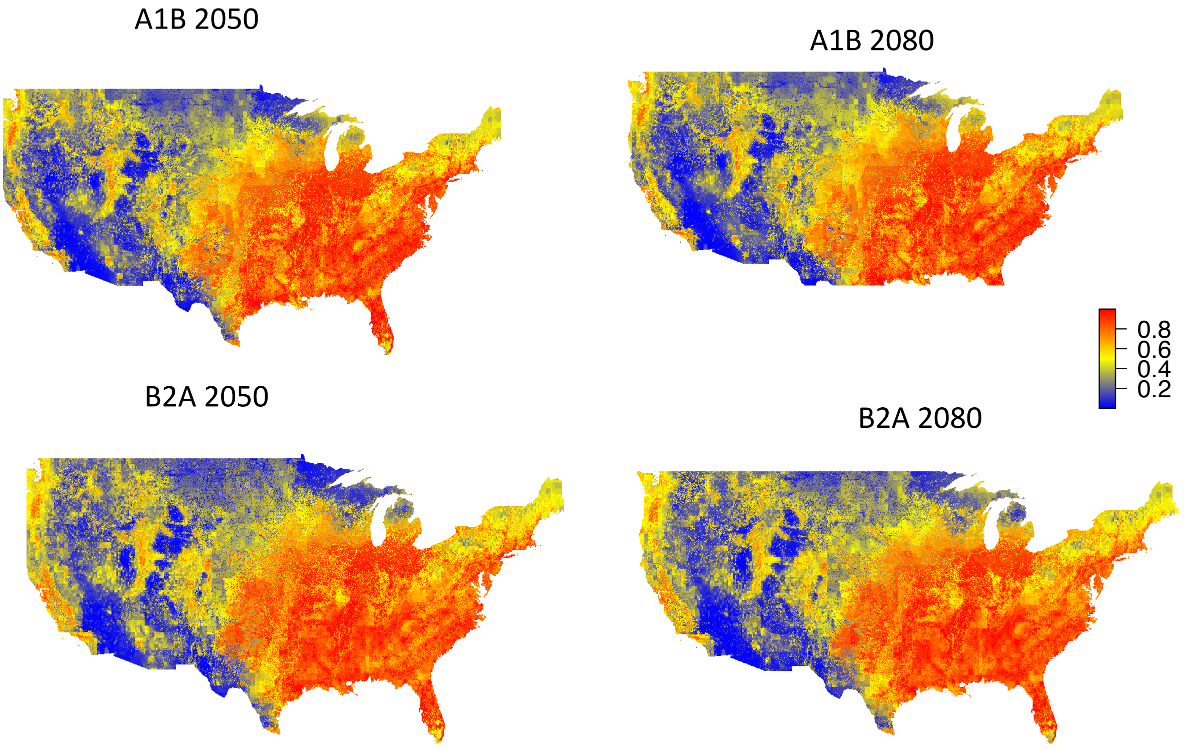

Figure S4: Cumulative impact using taxonomic diversity for all the endemic species (N=196) for each CO_2 emission scenarios and date. Overall, we observed a similar pattern of cumulative threat values across the emission scenarios and dates. Indeed, most of the high cumulative impact values are located on the eastern part of the USA while most of the medium to low cumulative values are located on the western part. However, we also observed some small differences between the two emission scenarios. In particular, high cumulative impact values (>0.7) covered a larger part of the western part of the US under the A1B scenario compared to the B2A emission scenario. In addition, the cumulative impact values are predicted to be more important (0.2 to 0.5) in the northern states like Washington, Montana, North Dakota under the A1B scenario compared to the B2A emission scenarios. Figure created in ArcGIS 10.2.1 software.

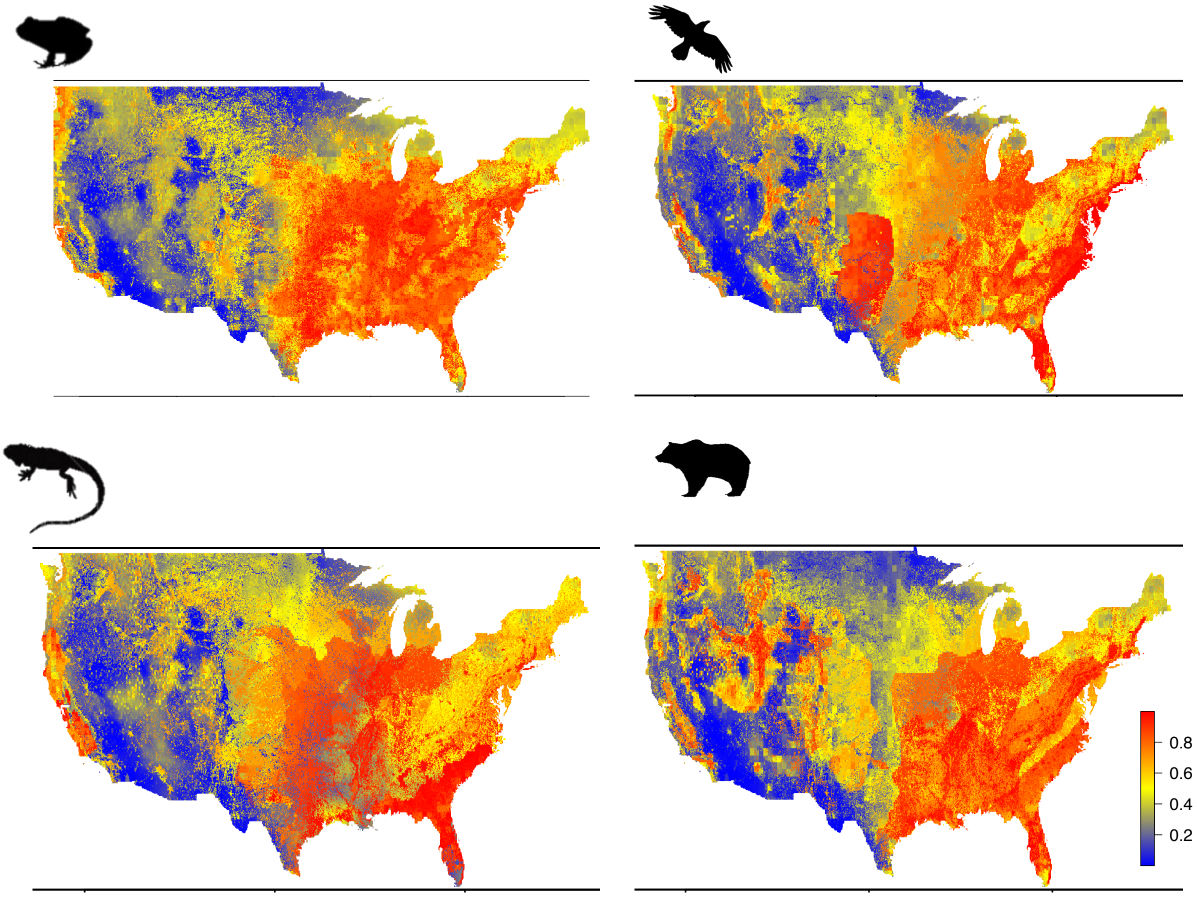

Figure S5: Cumulative impact maps using phylogenetic diversity for the A1B emission scenarios in 2080. The pattern of cumulative impact values using phylogenetic diversity is highly similar with Figure 3. Figure created in ArcGIS 10.2.1 software.

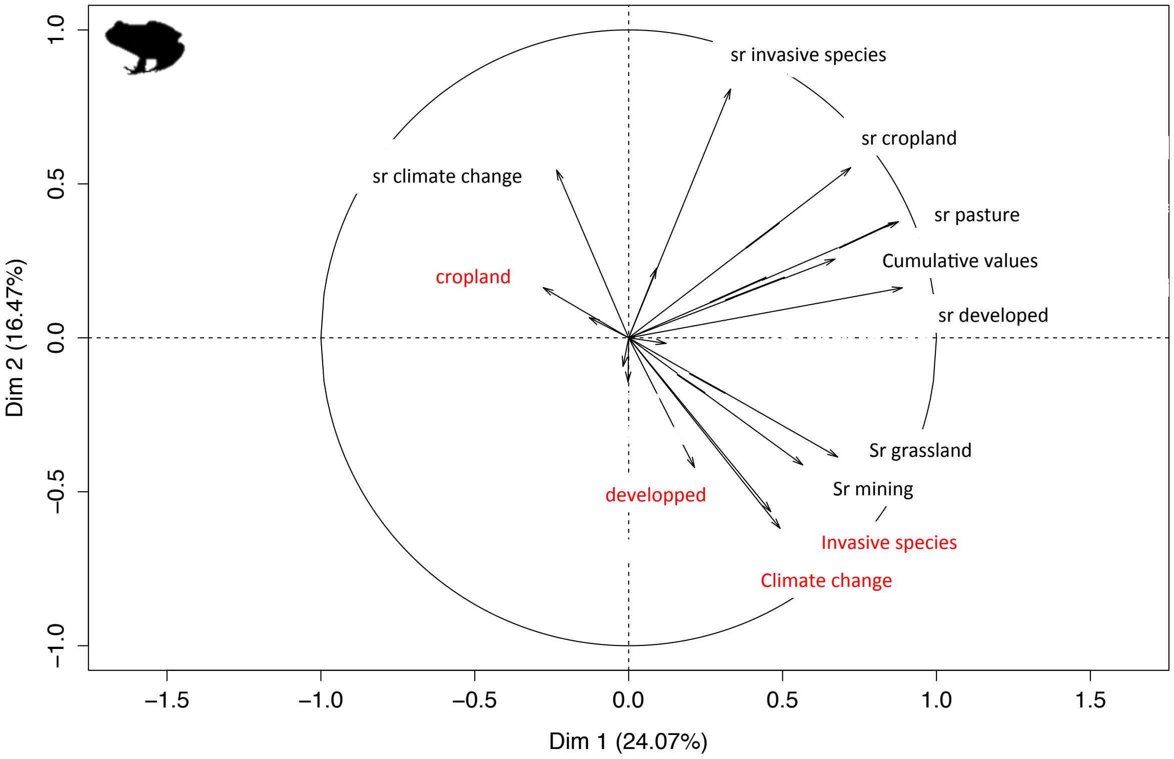

Figure S6. PCA biplot for amphibians taxa for the high cumulative impact values. Threats are in red. Abbreviation : *sr* represents species richness threatened by a given threat.

We performed principal components analyses (PCA) using the prcomp() function in R to assess whether the linear combinations of threat intensities and number of species vulnerable to these threats (species richness) could summarize most of the variation among high cumulative impact values.

Regarding amphibians, we found the first two components to account for 40.54% for the variation in cumulative impact values. Many of the same threats that had high correlations with the overall cumulative impact values (Figure 4) loaded highly on the first two PCA components, in particular number of species vulnerable to invasive species. Figure created in R 3.1.1.

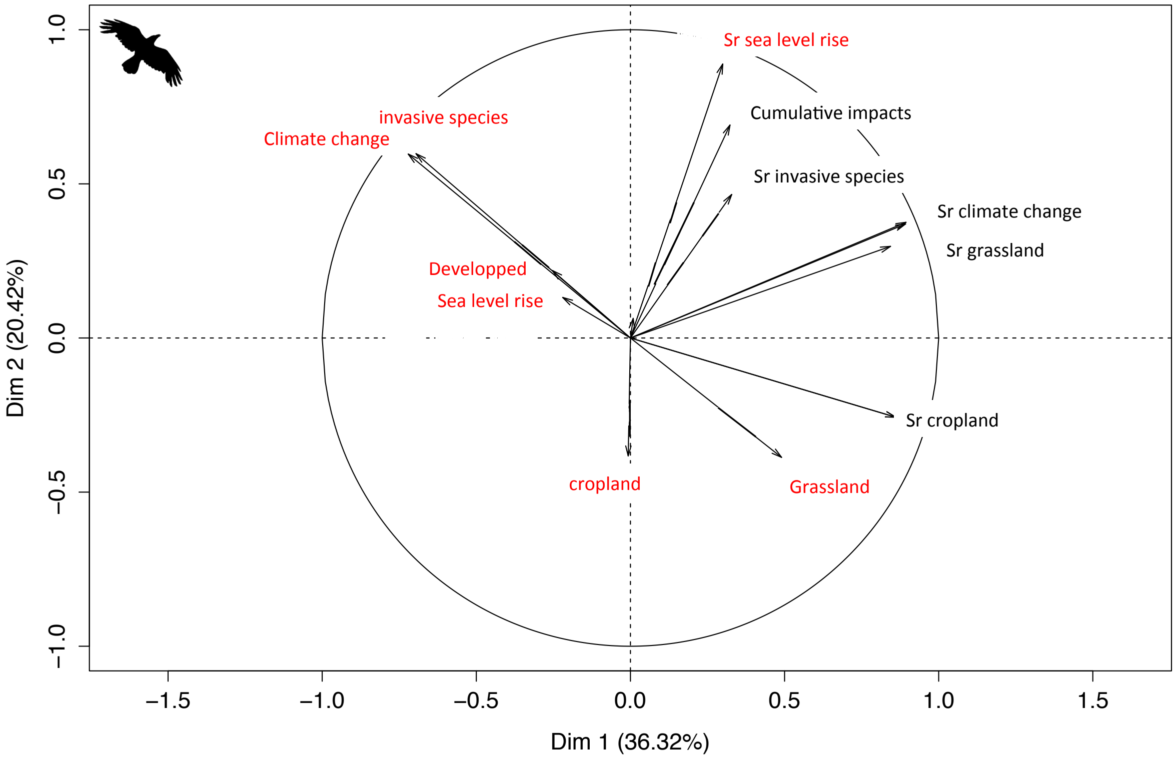

Figure S7. PCA biplot for birds taxa for the high cumulative impact values. For birds, we found the first two components to account for 56.74% for the variation in cumulative impact values. Variation in cumulative impacts was driven largely by concordant spatial patterns in multiple threats (*i.e.,* invasive species, climate change, *grassland, cropland*), and high species richness vulnerable to these threats. More specifically, *cropland* is highly associated with the first axis, while species richness components were more associated to the second axis. Figure created in R 3.1.1.

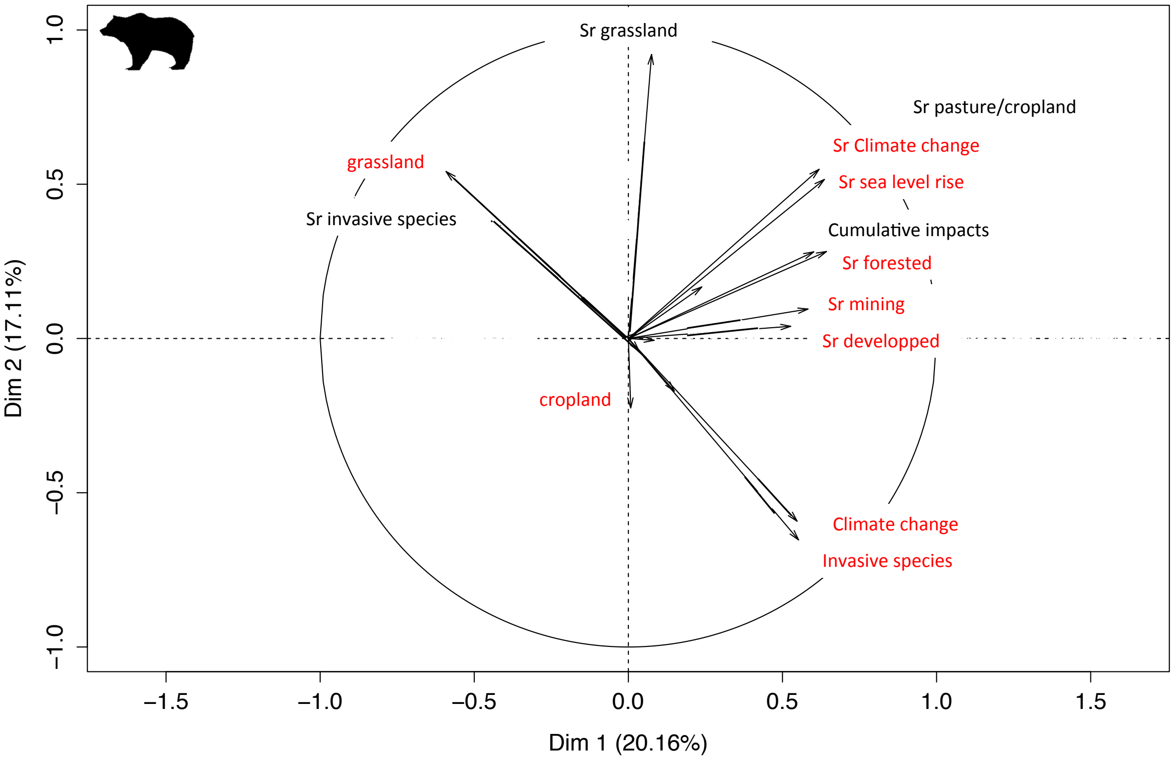

Figure S8. PCA biplot for mammals taxa for the high cumulative impact values. For mammals, we found the first two components to account for 37.37% for the variation in cumulative impact values. The variation explained by the first two PCA components was lower compared to the other taxonomic groups. Overall, all threats and species richness contributed to explain the variation among the cumulative impact values. Figure created in R 3.1.1.

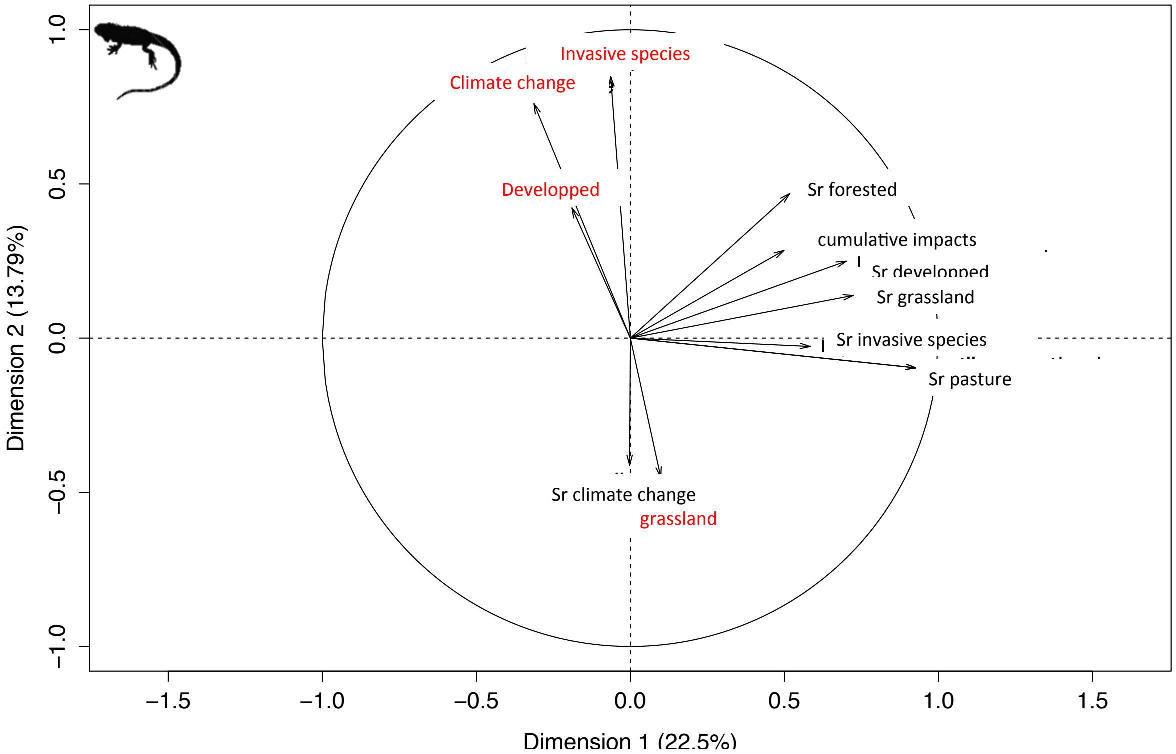

Figure S9. PCA biplot for reptiles taxa for the high cumulative impact values. We found the first two components to account for 36.29% for the variation in cumulative impact values. We found that most of the species richness variables were associated to the second PCA components, while the threats (climate change, invasive species, developed areas) were linked to the first PCA component. Figure created in R 3.1.1.

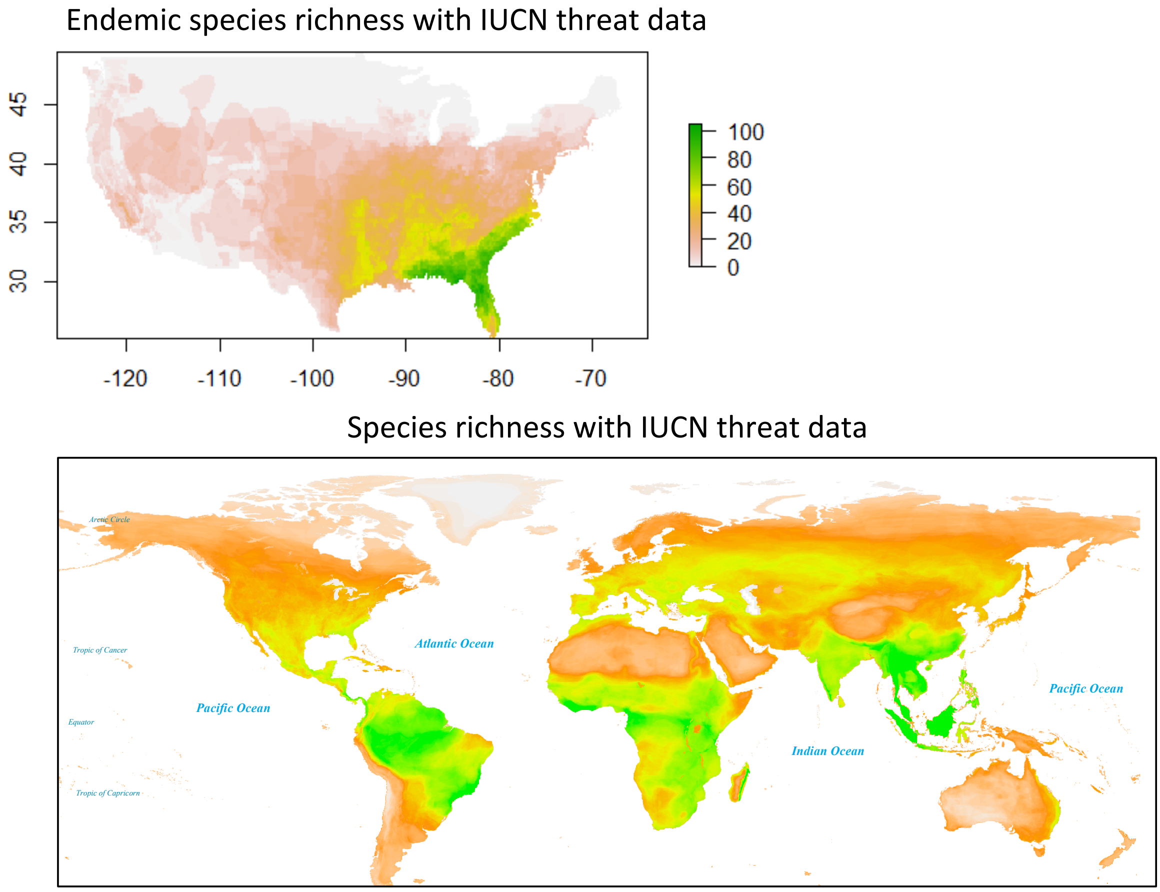

Figure S10. Species richness of endemic amphibians, birds, mammals, and reptiles that are evaluated by IUCN as vulnerable to at least one threat among the nine studied in this paper and species richness of amphibians, birds, mammals, and reptiles at the world scale. Figure created in R 3.1.1.

Suplementary material 1. Number of cumulative threat values exposure across endemic species mapped onto the phylogeny of amphibians and mammals.

	**Pagel's λ**	**Log-likelihood**	**Log-likelihood for lambda = 0**	***p-value***	
**Amphibians**	0.900	-63.446	-73.602	$6.580e^{-06}$	Phylosignal
**Birds**	$6.955e^{-05}$	-4.989	-4.989	1.00	No phylosignal
**Mammals**	0.701	-28.026	-35.242	$1.453e^{-04}$	Phylosignal
**Reptiles**	0.332	-26.651	-27.079	0.355	No phylosignal

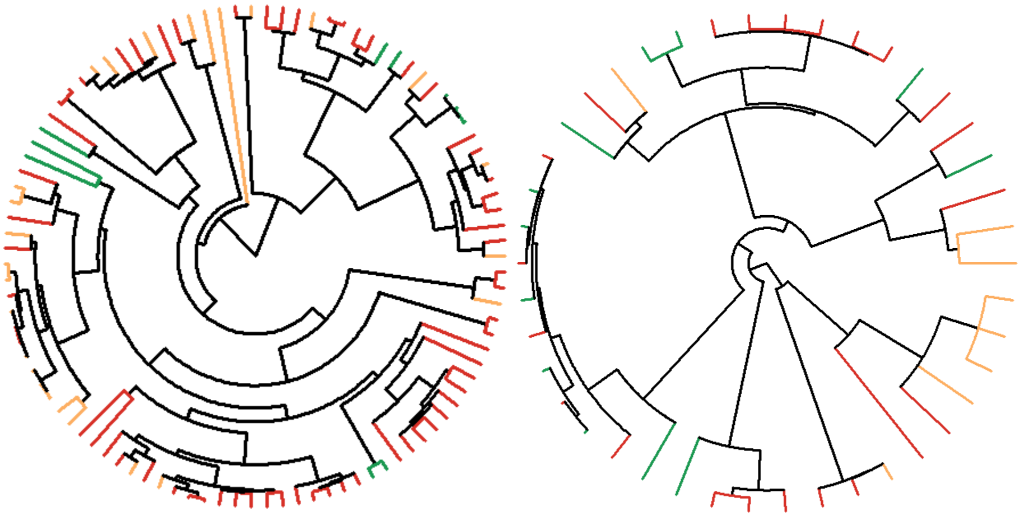

AMPHIBIANS **MAMMALS**

SI text.
SI 1: Detailed about the generation of threats and interpretation

Sea level rise

We determined the land area that will be permanently submerged by 1m and 2m sea-level rises, using the same methodology as in (C. Bellard, Leclerc, & Courchamp, 2014). Ongoing research suggests that future increase of global sea level rise will be 0.26 to 0.98 m for 2081-2100 (IPCC 2013). Several, recent studies strongly suggest that sea level rise could be substantially greater, i.e., an increase of 0.5 to 2.3 m by the end of the century (Grinsted, Jevrejeva, & S, 2010; Rahmstorf, 2007). Such increases could lead to the immersion of large portions of coastlines. The consequences of sea level rise for biodiversity might be significant, especially in the U.S. (Hinkel et al., 2014).
We considered two different sea level rise scenarios, ranging from 1 to 2m. This last scenario represents a realistic upper bound of sea level rise (Nicholls & Cazenave, 2010). More precise sea level rise scenarios (below the meter) are currently available. In addition, although sea level rise is likely to be heterogeneous, the regional variation in sea level rise is hard to predict. Therefore, we did not extrapolate heterogeneity, due to these high uncertainties (Nicholls & Cazenave, 2010). In addition, it is commonly accepted to use a homogeneous rise to assess the impact of sea level rise (Wetzel, Beissmann, Penn, & Jetz, 2013). We extracted elevation data from the digital elevation model developed by NASA's shuttle radar topography mission with 250 m resolution (Jarvis, Reuter, Nelson, & E. Guevara, 2008) across the United States. Potentially submerged areas include standing freshwater such as rivers, wetlands, and associated habitat connected to the ocean. We only considered flooded cells that connected to the ocean below a projected sea-level rise. This data layer presents for each cell a binary value of presence/absence of submersion by sea-level rise scenario.

Climate change

To determine areas that would experience climate change, we calculated the local climate change defined as the standardized Euclidean distance (SED) between the current and future climate data for each pixel (see formula below) using the same methodology as in (Céline Bellard et al., 2014). This approach assumes that the USA has specific climates accurately represented by the six selected climate variables. In addition, this approach also assumes that significant changes of climate will threaten species.

We used the methodology developed by Williams et al. (2007) to quantify the climate dissimilarity.

$$SED_i = \sqrt{\sum_{k=1}^{6} \frac{(a_{ki} - b_{ki})^2}{S_{ki}^2}} \qquad (1)$$

where a_{kI} and b_{ki} are the current and future means for climate variable k at grid point i, and S_{ki} is the standardized current climate value, following the methodology of Williams, Jackson, & Kutzbach, 2007). The standardization values were metrics of seasonality for temperature and precipitation variables. Standardizing each variable places all climate variables on a common scale (Veloz et al., 2012). We used six different climate variables linked to temperature and precipitation (i.e., annual mean temperature, maximum temperature of warmest month, minimum temperature of coldest month, annual precipitation, precipitation of wettest month and precipitation of driest month). Current climatic data were averaged from 1950-2000 data of the Worldclim database (Hijmans, Cameron, Parra, Jones, & Jarvis, 2005) and future climate data were obtained from the Global Climate Model data portal for 2050s (2040-2069) and 2080s (2070-2099) (Global Climate Model, 2013), all at 10' resolution (*i.e.,*

18.6 x 18.6 km). These variables were selected because they provide a combination of means, extreme and seasonality that are known to influence the distribution of species and were not collinear (Pairwise Pearson's r<0.75). Simulations of future climates were based on three general circulation models (i.e., HADCM3, CSIRO2 and CGCM2) and two CO_2 emission scenarios (i.e., B2A and A1B). This data layer presents continuous values of an index of local climate change per pixel.

Invasive alien species

We used projected threat of the 100 among the world's worst invasive alien species modeled by (C. Bellard et al., 2013) in 2050 and 2080 according to the A1B and B2A emissions scenarios at 0.5° resolution. This IUCN list of the "*100 of the world's worst invasive alien species*" provides the most geographically and taxonomically representative set of the most dangerous invasive alien species around the world, causing significant impacts on biodiversity and/or human activity in all ecosystems (Lowe, Browne, Boudjelas, & De Poorter, 2000).

Below is the methodology that have been used to generate this layer from (C. Bellard et al., 2013) .

Climate data. To characterize present-day climate, we used climatic data (averaged from 1950–2000) from the Worldclim database (Hijmans et al., 2005) at a 0.5° resolution. We selected six climatic variables: (i) mean diurnal range, (ii) maximum temperature of warmest month, (iii) minimum temperature of coldest month, (iv) annual precipitation, (v) precipitation of wettest month, and (vi) precipitation of driest month. In the case of freshwater species, many studies have revealed strong correlations between spatial patterns and climatic variables (Jocque, Field, Brendonck, & De Meester, 2010), mostly temperature and the availability of water, and have used species distributions mod- els to successfully predict the distribution of fishes (McNyset, 2005) and mussels (Drake & Bossenbroek, 2004). Future climate data were extracted from the Global Climate Model data portal (http://www.ccafs-climate.org/spatial_downscal- ing/). These models were statistically downscaled from the original Global Change Model outputs with the Delta method (Ramirez-Villegas & Jarvis, 2010). Due to large effects of different atmosphere ocean global circulation models (AOGCMs) on species range projections, simulations of future climate were based on three different AOGCMs (HADCM3, CSIRO2 and CGCM2) averaged from 2040 to 2069 ('2050') and 2070 to 2099 ('2080'). We used two different scenarios (A1B, B2A) that reflect different assumptions about demographic, socio- economic and technological development on greenhouse gas emission (Solomon et al., 2007).

Land use data. Current and future global land use and land cover variables were simulated by the Globio3 land model at a 0.5° resolution for two different emission scenarios A1B and B2A (Millennium Ecosystem Assessment et al., 2005; Alkemade et al., 2009). These data together with climate were used to model the potential distribution of the species in the 100 world's worst invasive species list. For the two selected emission scenarios, we re-classified the 30 different land cover types from the Globio3 data (Bartholomé & Belward, 2005) into 12 land cover type variables by grouping some of them together. These land use variables consisted of the proportion of the grid cell covered by (i) tree cover, (ii) tree cover regularly flooded, (iii) mosaic habitat, (iv) tree cover burnt, (v) shrub cover, (vi) herbaceous cover, (vii) cultivated and managed areas, (viii) bare areas, (ix) water bodies, (x) snow and ice, (xi) artificial surfaces and associated areas and (xii) pasture. We calculated for each pixel the proportion of each land cover type in 1970–2000 ('current'), '2050' and '2100'.

Invasive alien species data. We collected current distribution data for the species on the list 100 of the world's worst invasive alien species, compiled by the International Union for the Conservation of Nature (IUCN, Lowe et al., 2000). Developed in 2000 by the ISSG global network of over 1000 invasion biology experts, this synthesis included input from the wider community of practitioners and scientists with expertise on all taxonomic groups and environments. The list provides the most geographically and taxonomically representative set of the most dangerous invasive alien species around the world, causing significant impacts on biodiversity and/or human activity in all ecosystems. Data were collected from a variety of online databases, references and personal communications (see Bellard et al., 2013 for details).

Species distribution model projections

Modeling process. We modeled the potential distribution of the invasive species by combining available occurrences with a set of six climatic variables and 12 land use variables that we assumed to be important for invasive species. Analysis of the climate and land use preferences of a species can therefore be used to predict areas where the species could occur at global scales. Although other factors such as soil properties or micro-climate also determine the presence or absence of a species at the local scale, we assumed that climate and land use were the most important explanatory variables of species distribution at the global scale. We used six different SDMs, within the biomod v.2.0 platform (Thuiller, Lafourcade, Engler, & Araújo, 2009) carried out on the R platform. These models were: Generalized Linear Model, Generalized Boosting Trees, Multivariate Adaptive Regression Splines, Random Forest, Flexible Discriminant Analysis and Maximum Entropy. More details about the first five modeling techniques can be found in Thuiller et al., (2009) and in Elith et al., (2011) for Maximum Entropy. All models required presence and pseudo-absences (PAs or background). Five sets of PAs were generated by selecting from 1000 to 10 000 random points across the globe, according to the number of presences N (if $N \leq 1000$ then 1000 PAs were selected, else 10,000 PAs were selected) [as recommended by (Barbet-Massin, Jiguet, Albert, & Thuiller, 2012)]. Equal weightings were given to presences and PAs.

Evaluating model performance. We evaluated the predictive performance of each model using a repeated split sampling approach in which models were calibrated over 70% of the data and evaluated over the remaining 30%. This procedure was repeated four times. We used two different statistical met- rics: the true skill statistics (TSS) (Allouche, Tsoar, & Kadmon, 2006) and Area Under the ROC Curve (AUC) (Fielding & Bell, 1997). The TSS accounts for both omission and commission errors, and ranges from _1 to +1, where +1 indicates perfect agreement and 0 represents a random fit (Allouche et al., 2006). AUC values range from 0 to 1, and according to Swets (1988), an AUC above 0.8 is considered to have 'good' discrimination abilities. We also used the Boyce index to assess model performance (Boyce, Vernier, Nielsen, & Schmiegelow, 2002; Petitpierre et al., 2012). The Boyce index only requires presence data, where AUC and TSS require both presence and absence data. Boyce index measures how much model predictions differ from random distribution of the observed presences across the predic- tion gradients. Values of Boyce index vary between -1 and +1. Positive values indicate a model with present predictions that are consistent with the distribution of presences in the evaluation dataset; values close to zero mean that the model is not different from a random model. We calculated the Boyce index for each of the 60 models (GLM, MARS and MaXent) per species.

Ensemble modeling approach. The final calibration of every model for generating invasion scenarios used 100% of available data. We used an ensemble forecast approach to account for

the variability among the six species distribution models and the three general circulation models to get the central tendency (Araújo & New, 2007). To make sure no spurious models were used in the ensemble projections, we only kept the projections for which the model's evaluation estimated by AUC and TSS were higher than 0.8 and 0.6, respectively (e.g., Gallien, Douzet, Pratte, Zimmermann, & Thuiller, 2012), and a weight proportional to their TSS evaluation was associated with each model. Because of the potential problems raised by (Lobo, Jiménez-Valverde, & Real, 2008) on the use of AUC as a measure of model performance, we decided to use TSS for the final consensus distributions. The final current and future consensus distributions were obtained by calculating the weighted mean of the distribution for each scenario (Marmion, Parviainen, Luoto, & Heikkinen, 2009). This resulted in one current probability distribution map and three future probability distribution maps (because we used three global circulation models) for each emission scenario (A1B and B2A) and each species. Future probability maps were therefore averaged for each scenario. Then, we transformed the probability maps obtained from the ensemble projections into binary suitable/non-suitable maps using the threshold maximising the True Skill Statistics, as proposed by Allouche et al., (2006). This was done to ensure the most accurate predictions since it is based on both sensitivity and specificity (Jiménez-Valverde, 2012). We obtained one current binary distribution map and three future binary distribution maps per emission scenario and per species. Consensus binary maps were obtained attributing presence when the majority of GCMs (i.e., two of three) predicted presence, otherwise predicting absence.

Among these species modeled in Bellard et al., (2013), we have only kept 57 invasive alien species known to be already present in the United States including 18 terrestrial plants, ten terrestrial invertebrates, seven mammals, seven freshwater fishes, five aquatic invertebrates, four fungi, three amphibians, one aquatic plant, one bird, and one reptile, and 18 terrestrial plants. This data layer presents a continuous value of the number of invasive alien species per pixel.

Land-use change

We used projected land-use and land cover scenarios by 2050 and 2080 for two emission scenarios (i.e., B2A and A1B) developed by the United States Geological Survey using the FORecastingSCEnarios of future land-use (FORESCE) model at 250m resolution (Sleeter et al., 2013)). These spatially explicit models integrate both "topdown" drivers of land cover/land-use, such as demographic change, and local-scale "bottom-up" drivers such as biophysical site conditions (Sleeter et al., 2012). The USGS land use/land cover maps include 17 different classes, but we only considered six classes corresponding to the same classes regarding biodiversity threat (*i.e., Developed, Mining, Disturbed forested lands*, *Grassland*, *Cropland*, and *Pasture*). The table below represent the evolution of the six classes per scenarios and periods

Land_use	Period	Scenario	Percentage of area covered
	2000	-	2,45
	2050	A1B	4,57
**Developed**		B2A	3,37
	2080	A1B	5,39
		B2A	3,50
**Mining**	2000	-	0,10

	2050	A1B	0,16
		B2A	0,14
	2080	A1B	0,18
		B2A	0,15
**Forested lands**	2000	-	0,94
	2050	A1B	1,22
		B2A	1,08
	2080	A1B	1,52
		B2A	1,24
**Grassland**	2000	-	15,79
	2050	A1B	13,49
		B2A	15,81
	2080	A1B	12,00
		B2A	15,86
**Cropland**	2000	-	16,81
	2050	A1B	19,00
		B2A	15,26
	2080	A1B	20,66
		B2A	14,76
**Pasture**	2000	-	9,06
	2050	A1B	10,16
		B2A	8,31
	2080	A1B	10,99
		B2A	8,10

Table SI 1 : Evolution of the land use classes across time and emission scenarios.

SI 2: Detailed about the climate models and emission scenarios used in the study

We projected four main threats including sea level rise, climate change, invasive alien species and habitat changes (through six land use classes: Developed, Mining, Disturbed forested lands, Grassland, Cropland, and Pasture).

Choices of emission scenarios and global climate models.
Two IPCC emission scenarios were used for the projection of future climate for each threat (except sea level rise): A1B (maximum energy requirements, emissions balanced across fossil and non-fossil sources) and B2A (lower energy requirements and thus lower emissions than A1B). These two scenarios reflect different assumptions about demographic, socio-economic and technological development on greenhouse gas emission (Solomon et al., 2007). Regarding sea level rise, we used two different scenarios: increase by 1m and an increase of sea level rise by 2 meters. The 1m scenario followed the 1m scenario followed the current trend of sea level rise projection and was therefore similar to the B2A scenario. Conversely, the 2m scenario was similar to the A1B scenario. Therefore, we associated climate data, land use data, invasive alien species data according to the similarity between scenarios: A1B with 2m scenarios, and B2A with 1m scenario (see Table below).

Because of uncertainty in forecasting future distributions is largely related to global circulation models (Buisson, Thuiller, Casajus, Lek, & Grenouillet, 2010), we also used three global circulation models that simulated the impact of A1B and B2A emission scenarios on future climates. We chose the models that were available for both our climate scenarios, Hadley Centre Coupled Model version 3 (HADCM3), Coupled Global Climate Model (CGCM, A1B version 3.1 (t47), B2A; version 2) and the Commonwealth Scientific and Industrial Research Organization model (CSIRO, A1B: Mk3.0, B2A: Mk2). We chose two future periods: 2050s and 2080s (averaged from 2040 to 2069 and 2070 to 2099 respectively). These models were downscaled statistically using the Delta method (Ramirez-Villegas & Jarvis, 2010). The models were downloaded from the Global Climate Model data portal (http://www.ccafs-climate.org/).

	Projections for 2050s		Projections for 2080s	
**Sea level rise**	-	-	2m	1m
**Climate change** (ensemble of the 3 GCMs by calculating the mean of the 3 GCMs)	A1B	B2A	A1B	B2A
**Invasive alien species** (ensemble of the 3 GCMs by calculating the mean of the 3 GCMs)	A1B	B2A	A1B	B2A
**Land use changes**	A1B	B2A	A1B	B2A
**Cumulative threats/impacts**	2050s A1B	2050s B2A	2080s A1B	2080s B2A

Table SI 2 : Table of the scenarios available for each threat layer and how these scenarios were crossed for the final outputs.

The final cumulative threats and impacts values were calculated for 2050s and 2080s periods. Because sea level rise is likely to be less than 1 meter in 2050 and we do not have elevation data accuracy at less than 1 meter. We did not consider the sea level rise threat in the projections for 2050s period.

SI 3: Detailed about sensitivity tests of standardization methods.

Our approach requires merging intensity of threats and species richness of species vulnerable to these threats, the native units of which vary widely. Therefore, we rescaled the value per pixel of each variable to a 0-1 range to create a unitless relative metric for individual threat, species richness, and cumulative impact values.

We transformed these variables using three types of standardization. First, we used a natural-log transformation (ln[x+1]) before max-min rescaling, thereby compressing the upper end and expanding the lower end of the range. Secondly, we used a basic standardization, a max-min linear rescaling ($[x_i-x_{min}]/[x_{max}-x_{min}]$). Thirdly, we used a cumulative distribution function to replace each value with a score reflecting its percentile rank relative to all other pixels (Allan et al., 2013). The cumulative distribution function approach resulted in a uniform number of pixels at any given level of value, whereas the linear and log transformations allowed heterogeneity in the number of pixels at any given level of value.

In order to test the robustness of our results, we performed a sensitivity test to measure the sensitivity of our results to the standardization procedure by calculating spatial correlation between the different maps of cumulative impact values. To this aim, we analyzed five runs of 50,000 random pixels because it was not possible to calculate the spatial correlation metric for all the pixels. We used the cor() function in R. This method has already been used in several papers to test the robustness of the results using different standardization metric (Allan et al., 2013). We found that the spatial correlations were high among the resulting nine maps of cumulative impact (all taxa considered) [A1B 2080] based on pair-wise comparisons, Pearson's r: mean = 0.81 ± 0.08.

We conclude that our results about spatial patterns of cumulative impact values were independent of the normalization method. Therefore, the broad patterns were affected very little by the choice of transformation approach (e.g., figure below). Although the values distributed would not be exactly the same, the relative extreme cumulative impact values were similarly distributed across the country. Consequently, the choice of the standardization method can have consequences for resulting maps of cumulative impacts (except main patterns). There is no single right answer for how best to transform and normalize data (Halpern & Fujita, 2013). However, the choice of the approach should reflect relevance for the question of the paper. For all analyses presented in the article, we used the cumulative distribution function approach for graphical and interpretation purposes because the flat frequency distribution of cumulative impact values across pixels facilitates visualization of broad spatial patterns and allow to identify the relative highest areas of exposure, which are of high interest for the study.

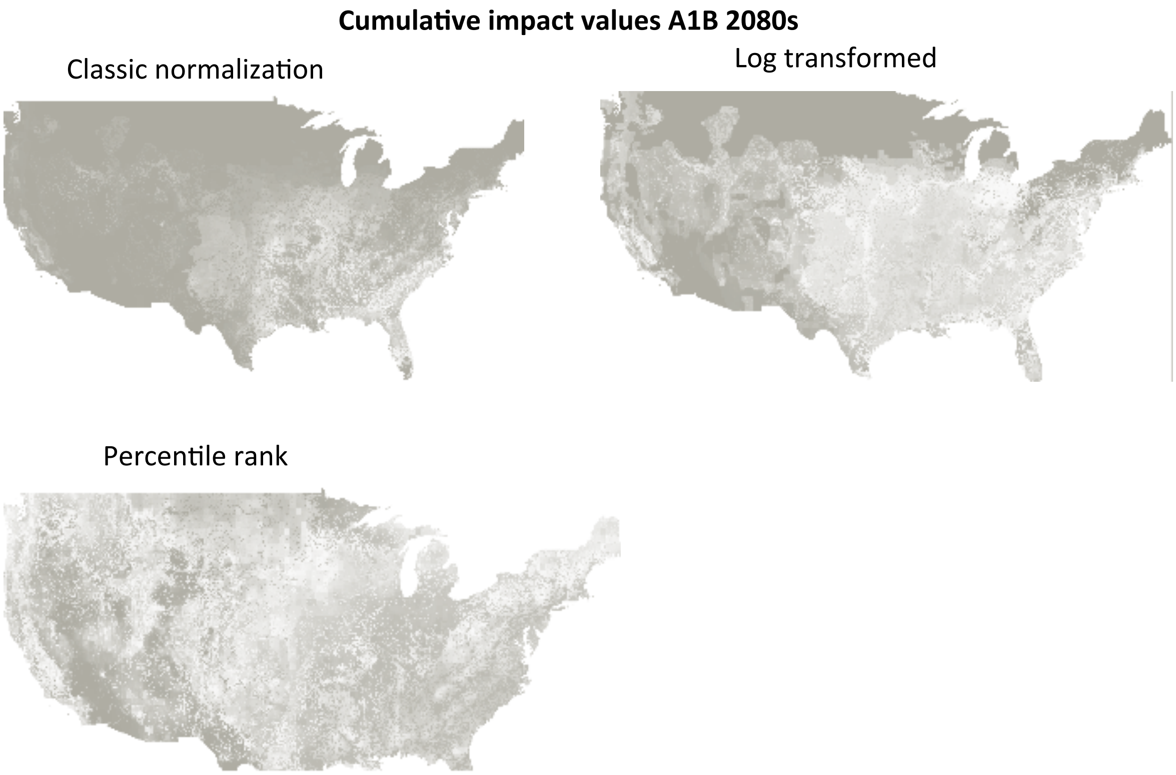

Figure SI3: Final maps of cumulative impact values under the A1B emission scenarios in 2080s for the three standardization methods: classic normalization, log transformed, and percentile rank. Figure created in R 3.1.1.

References :

Alkemade, R., Oorschot, M., Miles, L., Nellemann, C., Bakkenes, M., & ten Brink, B. (2009). GLOBIO3: A Framework to Investigate Options for Reducing Global Terrestrial Biodiversity Loss. *Ecosystems*, *12*(3), 374–390. doi:10.1007/s10021-009-9229-5

Allan, J. D., McIntyre, P. B., Smith, S. D. P., Halpern, B. S., Boyer, G. L., Buchsbaum, A., … Steinman, A. D. (2013). Joint analysis of stressors and ecosystem services to enhance restoration effectiveness. *Proceedings of the National Academy of Sciences of the United States of America*, *110*(1), 372–377. doi:10.1073/pnas.1213841110

Allouche, O., Tsoar, A., & Kadmon, R. (2006). Assessing the accuracy of species distribution models : prevalence , kappa and the true skill statistic (TSS). *Journal of Applied Ecology*, *46*, 1223–1232. doi:10.1111/j.1365-2664.2006.01214.x

Araújo, M. B., & New, M. (2007). Ensemble forecasting of species distributions. *Trends in Ecology & Evolution*, *22*(1), 42–7. doi:10.1016/j.tree.2006.09.010

Barbet-Massin, M., Jiguet, F., Albert, C. H., & Thuiller, W. (2012). Selecting pseudo-absences for species distribution models: how, where and how many? *Methods in Ecology and Evolution*, *3*(2), 327–338. doi:10.1111/j.2041-210X.2011.00172.x

Bartholomé, E., & Belward, A. S. (2005). GLC2000: a new approach to global land cover mapping from Earth observation data. *International Journal of Remote Sensing*, *26*(9), 1959–1977. doi:10.1080/01431160412331291297

Bellard, C., Leclerc, C., & Courchamp, F. (2014). Impact of sea level rise on the 10 insular biodiversity hotspots. *Global Ecology and Biogeography*, *23*(2), 203–212. doi:10.1111/geb.12093

Bellard, C., Leclerc, C., Leroy, B., Bakkenes, M., Veloz, S., Thuiller, W., & Courchamp, F. (2014). Vulnerability of biodiversity hotspots to global change. *Global Ecology and Biogeography*, n/a–n/a. doi:10.1111/geb.12228

Bellard, C., Thuiller, W., Leroy, B., Genovesi, P., Bakkenes, M., & Courchamp, F. (2013). Will climate change promote future invasions ? *Global Change Biology*, *19*(12), 3740–3748.

Boyce, M. S., Vernier, P. R., Nielsen, S. E., & Schmiegelow, F. K. A. (2002). E v aluating resource selection functions, *157*.

Buisson, L., Thuiller, W., Casajus, N., Lek, S., & Grenouillet, G. (2010). Uncertainty in ensemble forecasting of species distribution. *Global Change Biology*, *16*(4), 1145–1157. doi:10.1111/j.1365-2486.2009.02000.x

Drake, J. M., & Bossenbroek, J. M. (2004). The Potential Distribution of Zebra Mussels in the United States. *BioScience*, *54*(10), 931. doi:10.1641/0006-3568(2004)054[0931:TPDOZM]2.0.CO;2

Elith, J., Phillips, S. J., Hastie, T., Dudı, M., Dudík, M., Chee, Y. E., & Yates, C. J. (2011). A statistical explanation of MaxEnt for ecologists. *Diversity and Distributions*, *17*(1), 43–57. doi:10.1111/j.1472-4642.2010.00725.x

Fielding, A. H., & Bell, J. F. (1997). A review of methods for the assessment of prediction errors in conservation presence/absence models. *Environmental Conservation*, *24*(1), 38–49. doi:10.1017/S0376892997000088

Gallien, L., Douzet, R., Pratte, S., Zimmermann, N. E., & Thuiller, W. (2012). Invasive species distribution models - how violating the equilibrium assumption can create new insights. *Global Ecology and Biogeography*, *21*(11), 1126–1136. doi:10.1111/j.1466-8238.2012.00768.x

Global Climate Model. (2013). Retrieved from (http://www.ccafs-climate.org/spatial_downscaling/

Grinsted, A., Jevrejeva, J. C., & S, M. (2010). Reconstructing sea level from paleo and projected temperatures, 461–472. doi:10.1007/s00382-008-0507-2

Halpern, B. S., & Fujita, R. (2013). Assumptions, challenges, and future directions in cumulative impact analysis. *Ecosphere*, *4*(10), 1–11.

Hijmans, R. J., Cameron, S. E., Parra, J. L., Jones, P. G., & Jarvis, A. (2005). Very high resolution interpolated climate surfaces for global land areas. *International Journal of Climatology*, *25*(15), 1965–1978. doi:10.1002/joc.1276

Hinkel, J., Lincke, D., Vafeidis, a. T., Perrette, M., Nicholls, R. J., Tol, R. S. J., … Levermann, A. (2014). Coastal flood damage and adaptation costs under 21st century sea-level rise. *Proceedings of the National Academy of Sciences*, *111*(9), 3293–3297. doi:10.1073/pnas.1222469111

Jarvis, A., Reuter, H. I., Nelson, A., & E. Guevara. (2008). Hole-filled SRTM for the globe Version 4, available from the CGIAR-CSI SRTM 90m Database.

Jiménez-Valverde, A. (2012). Insights into the area under the receiver operating characteristic curve (AUC) as a discrimination measure in species distribution modelling. *Global Ecology and Biogeography*, *21*(4), 498–507. doi:10.1111/j.1466-8238.2011.00683.x

Jocque, M., Field, R., Brendonck, L., & De Meester, L. (2010). Climatic control of dispersal-ecological specialization trade-offs: a metacommunity process at the heart of the latitudinal diversity gradient? *Global Ecology and Biogeography*, *19*(2), 244–252. doi:10.1111/j.1466-8238.2009.00510.x

Lobo, J. M., Jiménez-Valverde, A., & Real, R. (2008). AUC: a misleading measure of the performance of predictive distribution models. *Global Ecology and Biogeography*, *17*(2), 145–151. doi:10.1111/j.1466-8238.2007.00358.x

Lowe, S., Browne, M., Boudjelas, S., & De Poorter, M. (2000). *100 of the World's Worst Invasive Alien Species A selection from the Global Invasive Species Database.* (N. Speciali, Ed.) (p. 12). Invasive Species Specialist Group.

Marmion, M., Parviainen, M., Luoto, M., & Heikkinen, R. K. (2009). Evaluation of consensus methods in predictive species distribution modelling, 59–69. doi:10.1111/j.1472-4642.2008.00491.x

McNyset, K. M. (2005). Use of ecological niche modelling to predict distributions of freshwater fish species in Kansas. *Ecology of Freshwater Fish*, *14*(3), 243–255. doi:10.1111/j.1600-0633.2005.00101.x

Nicholls, R. J., & Cazenave, A. (2010). Sea-level rise and its impact on coastal zones. *Science (New York, N.Y.)*, *328*(5985), 1517–20. doi:10.1126/science.1185782

Petitpierre, B., Kueffer, C., Broennimann, O., Randin, C., Daehler, C., & Guisan, A. (2012). Climatic niche shifts are rare among terrestrial plant invaders. *Science (New York, N.Y.)*, *335*(6074), 1344–8. doi:10.1126/science.1215933

Rahmstorf, S. (2007). A semi-empirical approach to projecting future sea-level rise. *Science (New York, N.Y.)*, *315*(5810), 368–70. doi:10.1126/science.1135456

Ramirez-Villegas, J., & Jarvis, A. (2010). *Downscaling Global Circulation Model Outputs: The Delta Method Decision and Policy Analysis Working Paper No.1.*

Sleeter, B. M., Sohl, T. L., Bouchard, M. A., Reker, R. R., Soulard, C. E., Acevedo, W., … Zhu, Z. (2012). Scenarios of land use and land cover change in the conterminous United States: Utilizing the special report on emission scenarios at ecoregional scales. *Global Environmental Change*, *22*(4), 896–914. doi:10.1016/j.gloenvcha.2012.03.008

Sleeter, B. M., Sohl, T. L., Loveland, T. R., Auch, R. F., Acevedo, W., Drummond, M. A., … Stehman, S. V. (2013). Land-cover change in the conterminous United States from 1973 to 2000. *Global Environmental Change*, *23*(4), 733–748. doi:10.1016/j.gloenvcha.2013.03.006

Solomon, S., Qin, D., M. Manning, Z. Chen, M. Marquis, K.B. Averyt, … H.L. Miller. (2007). *Contribution of Working Group I to the Fourth Assessment Report of the Intergovernmental Panel on Climate Change* (p. 996). United Kingdom and New York, NY, USA.

Swets, J. A. (1988). Measuring the accuracy of diagnostic systems. *Science*, *240*(4857), 1285–1293. Retrieved from http://www.sciencemag.org/content/240/4857/1285.abstract

Thuiller, W., Lafourcade, B., Engler, R., & Araújo, M. B. (2009). BIOMOD - a platform for ensemble forecasting of species distributions. *Ecography*, *32*(3), 369–373. Retrieved from http://doi.wiley.com/10.1111/j.1600-0587.2008.05742.x

Veloz, S. D., Williams, J. W., Blois, J. L., He, F., Otto-Bliesner, B., & Liu, Z. (2012). No-Analog Climates and Shifting Realized Niches During the Late Quaternary: Implications for 21st-Century Predictions by Species Distribution Models. *Global Change Biology*, *18*(5), 1698–1713. doi:10.1111/j.1365-2486.2011.02635.x

Wetzel, F. T., Beissmann, H., Penn, D. J., & Jetz, W. (2013). Vulnerability of terrestrial island vertebrates to projected sea-level rise. *Global Change Biology*, *19*(7), 2058–70. doi:10.1111/gcb.12185

Williams, J. W., Jackson, S. T., & Kutzbach, J. E. (2007). Projected distributions of novel and disappearing climates by 2100 AD. *Proceedings of the National Academy of Sciences of the United States of America*, *104*(14), 5738–42. doi:10.1073/pnas.0606292104
